# Computer-Aided Design of A-Trail Routed Wireframe DNA Nanostructures with Square Lattice Edges

**DOI:** 10.1021/acsnano.2c11982

**Published:** 2023-03-23

**Authors:** Marco Lolaico, Sebbe Blokhuizen, Boxuan Shen, Yang Wang, Björn Högberg

**Affiliations:** † Department of Medical Biochemistry and Biophysics, Karolinska Institutet, SE-17177 Stockholm, Sweden; ‡ Biohybrid Materials, Department of Bioproducts and Biosystems, Aalto University School of Chemical Engineering, P.O. Box 16100, 00076 Aalto, Finland

**Keywords:** DNA nanotechnology, scaffolded DNA origami, wireframe origami, four-helix bundle, coarse-grained molecular dynamics

## Abstract

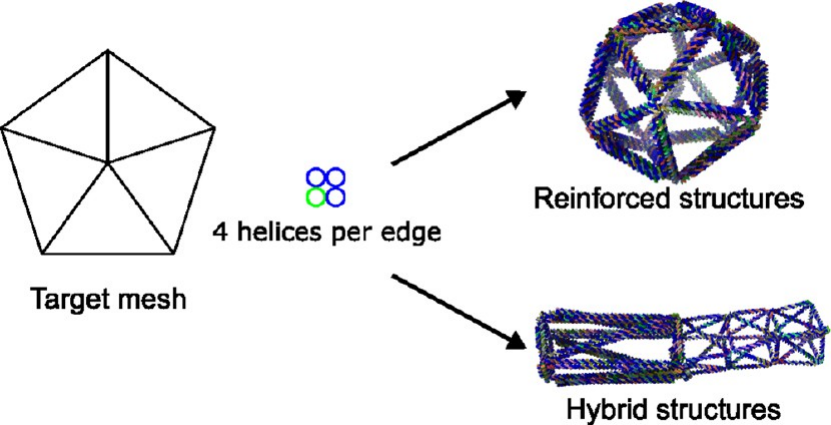

In recent years, interest in wireframe DNA origami has increased, with different designs, software, and applications emerging at a fast pace. It is now possible to design a wide variety of shapes by starting with a 2D or 3D mesh and using different scaffold routing strategies. The design choices of the edges in wireframe structures can be important in some applications and have already been shown to influence the interactions between nanostructures and cells. In this work, we increase the alternatives for the design of A-trail routed wireframe DNA structures by using four-helix bundles (4HB). Our approach is based on the incorporation of additional helices to the edges of the wireframe structure to create a 4HB on a square lattice. We first developed the software for the design of these structures, followed by a demonstration of the successful design and folding of a library of structures, and then, finally, we investigated the higher mechanical rigidity of the reinforced structures. In addition, the routing of the scaffold allows us to easily incorporate these reinforced edges together with more flexible, single helix edges, thereby allowing the user to customize the desired stiffness of the structure. We demonstrated the successful folding of this type of hybrid structure and the different stiffnesses of the different parts of the nanostructures using a combination of computational and experimental techniques.

DNA nanotechnology and, in particular, the DNA origami method have been demonstrated to be a versatile construction technique to enable the fabrication of complex 2D and 3D nanoscale structures.^1−6^ In this approach, a long, single-stranded *scaffold* strand of DNA is hybridized to complementary shorter oligonucleotides (*staples*) to create the target structure. The first DNA origami nanostructures were based on lattices,^2^ i.e., honeycomb^3^ and square^7^ lattices, helped by the subsequent development of the software caDNAno.^8^ DNA origami has found use in a wide variety of fields that range from physics plasmonics^9−12^ to drug delivery,^13−15^ as well as use as a precision tool in basic life science research.^16−19^

In subsequent years, the field has expanded with the introduction of methods and software for the design of wireframe DNA nanostructures.^20−27^ Since manual scaffold routing in wireframe DNA origami is much more challenging than for standard lattice-based structures, most of the design for wireframe DNA origami is carried through automatic or semiautomatic top-down software. These software packages, generally, take a 2D or 3D mesh as input, perform the routing of the scaffold on the selected mesh, and in the end, return the design staple sequences required for the design of the DNA origami structure that can be ordered and synthesized. The scaffold routings that have been used to create wireframe DNA origami from target meshes can be divided in two main categories: either A-trail routings^22,25,28^ or spanning tree routings^24,29,30^ (Supplementary Figure S1).

Wireframe DNA origami present different characteristics when compared with lattice-based structures. They are generally more material-efficient and use less scaffold for the same area (for 2D structures) or volume (for 3D structures). In addition, they tend to natively have a higher stability than lattice-based structures in low-cationic buffers, such as physiological buffers, probably because of the lower density of DNA. Moreover, it has been shown that wireframe structures interact differently than lattice-based ones with cellular systems: recently, we showed that wireframe DNA origami can more effectively penetrate cell spheroid tissue models.^31^

Another consequence of the lower density of DNA in the edges of wireframe DNA origami is a lower rigidity of the edges. The early automated design methods for wireframe DNA origami were focused on design with edges of up to two helices per edge, which created mechanically compliant structures. This can be a drawback for certain applications, and it has been addressed in different ways: in the DAEDALUS^23^ and the METIS^27^ design approaches, six-helix bundles (6HB) are used to represent the edges of structures based on spanning tree routing, which significantly improves the stiffness. In a different approach, we also tried to improve the stiffness of A-trail routed structures created with the BSCOR-vHelix design by modifying the design choices^32^ or by improving the structures by evolving the structures *in silico*.^33^

Here, we increase the repertoire of A-trail routed wireframe DNA origami nanostructures by using a four-helix bundle (4HB). We choose 4HB because they have been shown to have higher stiffness than single DNA helices.^34^ To transform the single helices into 4HB in A-trail routed scaffold designs, we add an additional scaffold loop in the helices representing the edges of the structures, which introduces two additional helices. The fourth helix in the bundle is composed of mini-scaffolds, which are additional short synthetic strands of DNA acting like the scaffold.^35−37^ We provide evidence that the structures can be folded with a high degree of fidelity to the original designs. Furthermore, we demonstrate their enhanced stiffness compared with nanostructures with a single helix as edges. In addition, we show that the reinforced edges can be selectively introduced together with nonreinforced single helices in a single structure, thereby creating “hybrid structures” with segments with a varied helix count and, thus, stiffness in the same nanoparticle. For a broader dissemination of our approach, we also designed a graphical user interface (GUI) that allows a streamlined workflow from a target mesh to the complete design whereby the user can freely select which edges to be reinforced or not.

## Results and Discussion

### Scaffold Routing and Staples Design

**Figure 1:**
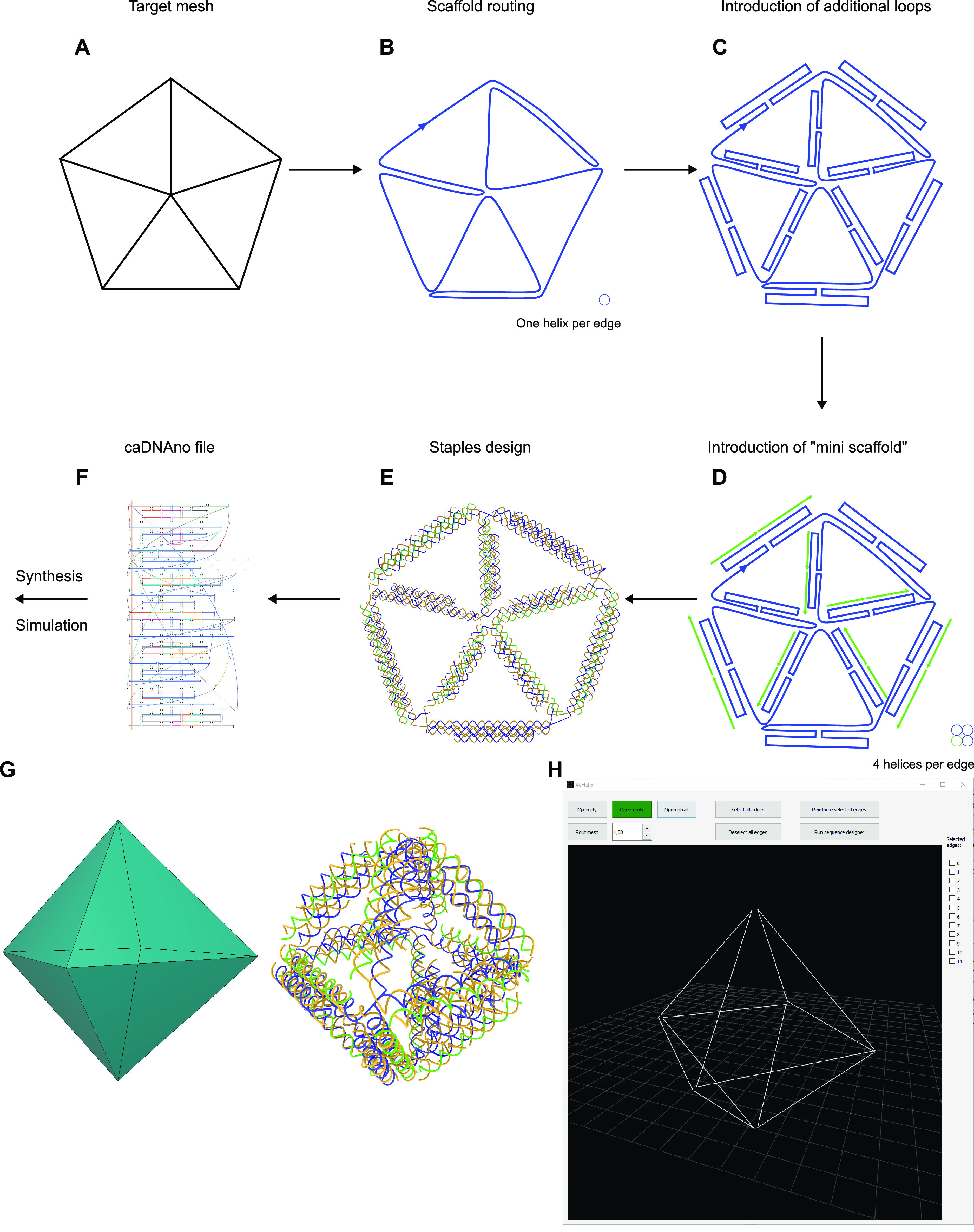
Overview of the design procedure for 4HB-reinforced structures. (A) The target mesh (in this case, a 2D pentagon) is defined as a set of vertices and faces. (B) The first step in the design procedure is to create the A-trail scaffold routing, with one helix per edge and some helper “double edges” to make the mesh Eulerian. (C) The loops are added in the scaffold routing, with one or more crossover connecting them to the main helix (here, only one crossover is present for the sake of clarity). (D) The mini-scaffolds are then added in the reinforced single edges. The mini-scaffolds are short (30–60 bp) and not part of the main scaffold. (E) The staples are added on the basis of the scaffold routing. The staples in the bundles are generally 32 bp long with a seed of 16 bp. (F) The output of the design procedure is a caDNAno file that can be easily modified and used to order staples and mini-scaffolds for synthesis of the nanoparticles or simulations. (G) Example of design of a 3D nanostructure from a 3D mesh. (H) A screenshot of the GUI that can be used to design reinforced and hybrid nanostructures.

Two-dimensional and three-dimensional geometries can be described as wireframe meshes in simple text format using the set of vertices and faces that compose it. This simple wireframe geometry can be used to define the target shape for DNA-based nanostructures (Figure 1A). In this work, the routing of the scaffold is based on the previously published A-trail method (implemented in the BSCOR/vHelix software suite).^22,25^ The routing of the scaffold through the mesh is based on A-trail, a type of Eulerian circuits where consecutive edges are always neighbors in the ordering around the vertices. In this procedure, almost every edge of the structure is represented by a single DNA helix, while some “double edges” might need to be added in some positions to make the mesh Eulerian. Once the routing is found, the staples are added, and the structure goes through a physical relaxation procedure to ensure minimal strain.

After the first routing is completed, most of the edges of the mesh are represented by a single helix (Figure 1B). At this point, two additional scaffold helices are added to each edge. These helices are connected through one or more double-crossovers to the scaffold, which forms a loop (Figure 1C). After this is done, a third helix (the fourth helix in the 4HB) is added: this helix is composed of additional mini-scaffolds,^35,36^ short sequences not connected to the main scaffold (Figure 1D). The automatic generation of the helices follows three criteria. First, the helices are added only on the edges that should be reinforced, and only if the edge has more than around 30 bases, in order to allow for a stable loop to be extruded from the scaffold. Second, in the case of an edge that was originally a double edge, only the two helices connected to the scaffold are added: in this case, the 4HB will be formed by the double edge and the two scaffold loops. Third, the length of the additional helices is derived from the length of the main edge: the two helices of the loop are slightly shorter than the main edge (limited by the possible location of crossovers) to avoid clashes between helices in the vertices, while the mini-scaffold helix has the same length as the main helix. The mini-scaffold pieces themselves are oligos of between 30 and 60 bp in order to comply with standard oligonucleotide synthesis.

Once the routing of the scaffold is completed, the design of the complementary staples can be determined (Figure 1E). The staples can be divided into two types: the ones connecting adjacent edges at the vertices and the ones connecting helices within each 4HB. The routing of the staples of the first kind is based on the BSCOR routing, and their length varies, generally between 30 and 60 bp. For the second type of staple, the routing is modular, and the length is designed to be 32 bases with a single 16 bases seed.^35^ In the vertices of the structures, we add a few unpaired bases to fill the gaps between the two helices to account for possible imprecisions in the relaxation. This applies to both the scaffold and the staples: in the staples, these unpaired bases are generally Ts. The addition of these unpaired bases allows the software to create structures with edges of any lengths without being limited by the number of bases in a helical turn.

The final structure is then converted into a caDNAno JSON file, which allows for finer modifications to the structure and use for further import into simulation and sequence generation for synthesis.

To facilitate the design of the reinforced structures, we implemented the software with a GUI. This allows the user to easily import meshes, decide which edges should be reinforced, and obtain the caDNAno file of the design. The 2D and 3D target geometries are specified by the user using a polygonal mesh in PLY format. Once the PLY is loaded, the necessary files for the routing are generated. At this point, the user can decide which edges to reinforce. The target mesh and the scaffold routing can be visualized in the GUI. After the routing is determined, the software generates a caDNAno JSON file, thereby allowing modifications to the structure. The GUI can also be used to process the modified caDNAno file to produce files containing the sequences of staples and mini-scaffolds (Figure 1F).

### Structures Reinforced with Four-Helix Bundles

To evaluate the design procedure, we used it to generate a library of 2D and 3D DNA nanostructure designs with different geometries, different lengths of the 4HB edges, and different vertex angles. The folding of all the structures was optimized for salt concentration, staples concentration, and annealing times, as evaluated by agarose gel electrophoresis (AGE) (Supplementary Figures S17–S22) and transmission electron microscopy (TEM) (Supplementary Figures S2–S16). The folding yield was also estimated from the AGE and ranged from 5% to 85% depending on the structure design (Supplementary Table 2).

**Figure 2:**
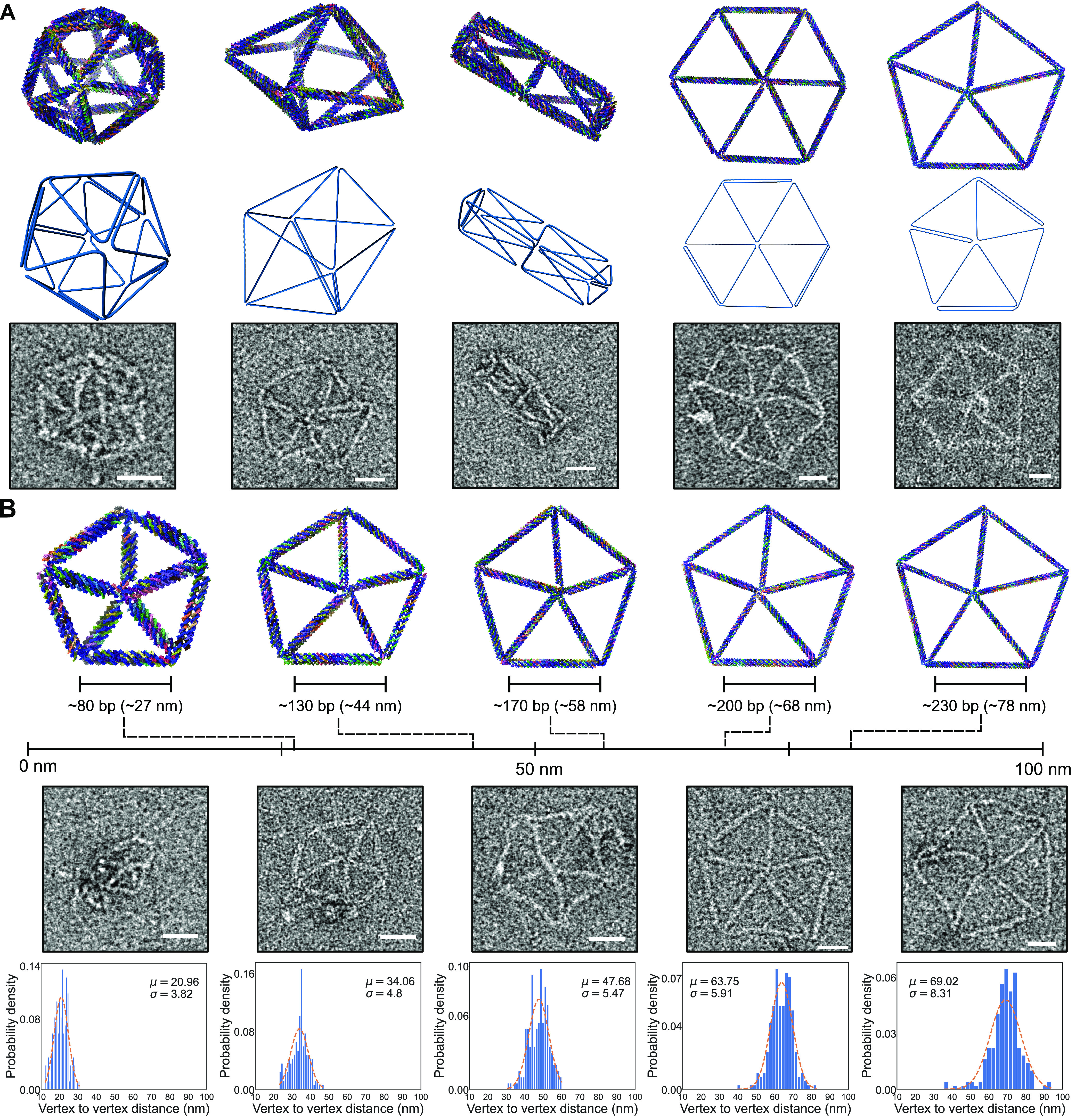
(A) Validation of the folding of reinforced structures. Top row: renders of the five designed structures, including an icosahedron, a pentagonal bipyramid, a reinforced rod, a 2D hexagonal mesh, and a 2D pentagonal mesh. Middle row: render of the scaffold routing, where only the original A-trail routing is shown for clarity. Bottom row: negative staining TEM of the successfully folded structures. Scale bars are 20 nm. (B) The 4HB structures with increasing edge lengths. The edge length in this 2D pentagonal mesh increases from around 80 bp to around 230 bp. Top row: renders of the designs, including 4HB reinforcements with increasing edge lengths. Middle row: negative staining EM pictures of the different 2D structures. Bottom row: plot of the vertex-to-vertex distance in the different structures. Scale bars are 20 nm.

The 3D structures we designed are an octahedron, an icosahedron, a pentagonal bipyramid, and a square-based rod (Figure 2A). The icosahedron has edges of around 100 bp (ca. 34 nm long). The structure appears (from TEM data, Supplementary Figures S9 and S12) to have good structural stability and maintain the designed shape. The pentagonal bipyramid has longer edges of around 180 bp that seem to bend more than the edges of the icosahedron. The next structure we designed is a reinforced rod, a type of structure that has been previously well studied.^31−33^ This rod has a high degree of diversity in the length of the edges, which range from around 50 bp to around 150 bp. The structure can fold with a high fidelity to the initial design, as judged by the TEM images (Figure 2A and Supplementary Figures S7 and S14). We also designed and synthesized different 2D structures (Figure 2A). We created a pentagonal and a hexagonal mesh with edges of up to around 270 bp and 200 bp, respectively. The structures were characterized using TEM, and the images show monodisperse, well-folded structures.

Next, we focused on the pentagonal mesh structure to study the behavior of the reinforced edges at different length scales. We designed different versions of this structure, where the only difference was the length of the edges (Figure 2B). These designs comprised five pentagonal meshes, with edge lengths ranging from around 80 bp (27 nm) to around 230 bp (78 nm). From the TEM results, we can observe well-folded nanostructures for all five of the designs. It is possible to observe the presence of an unfolded scaffold in the smaller scale structures that sometimes deforms the shape of the structure. The edges of the structures look rigid in the images, which suggests that the reinforced edges are still rigid up to the maximal length tested here. We quantified the vertex-to-vertex distances from TEM images and found a consistent increase in this distance with the increase in the designed edge lengths, as expected in the case of properly formed rigid edges (Figure 2B). In agarose gel (Supplementary Figures S17–S22), it is possible to see a trend where structures with longer edges show more unwanted multimerization and aggregation in the pockets. This trend is particularly evident for the pentagonal mesh structures. We attribute this to the fact that long, rigid edges might increase the contact order of staples at the vertices and, thus, negatively affect the folding dynamics.^35^ This seems to be dependent on factors like the folding program, since the assembly yield can be improved by optimizing the folding program (Supplementary Figures S17 and S18).

**Figure 3:**
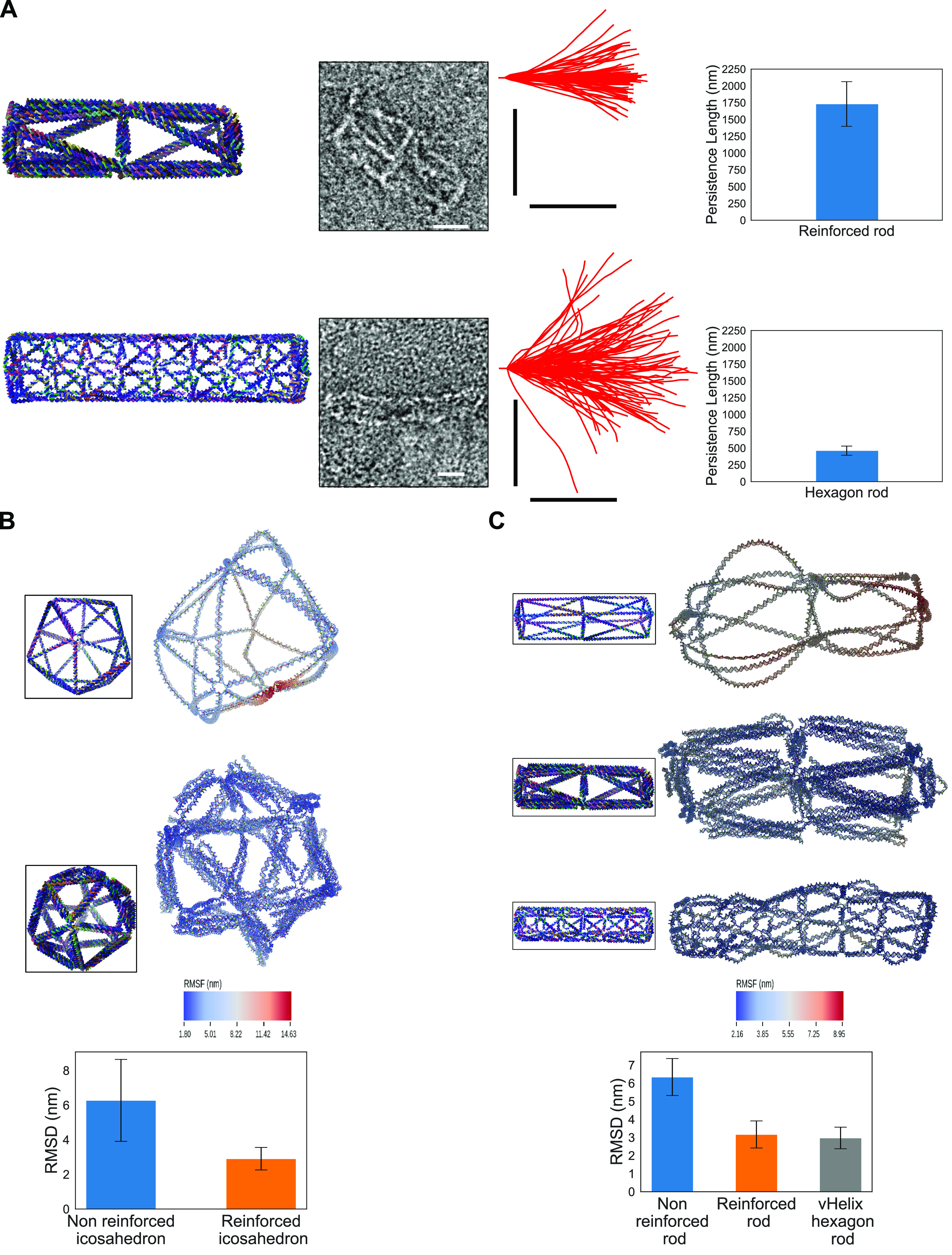
(A) Persistence length comparison between the reinforced rod and the hexagon rod with a single helix per edge. First column: renders of the designs of the structures. Second column: example of the TEM images used to estimate the persistence length of the structures. Third column: Trajectories of the structures extracted from the TEM images. Fourth column: plots of estimated persistence length. Scale bars are 20 nm. (B,C) Results of oxDNA simulations of nonreinforced vs reinforced structures. (B) On the top, comparison between the nonreinforced and the reinforced version of the icosahedron. The computed mean structure and RMSF are depicted. In the inset are the structures as designed. On the bottom, plotted RMSD of the two structures. (C) On the top, comparison of the nonreinforced rod, reinforced rod, and a hexagon rod of a similar length and diameter. The computed mean structure and RMSF are depicted. In the inset are the structures as designed. On the bottom, plotted RMSD of the three structures.

Next, we analyzed and compared the rigidity between the reinforced structures and the nonreinforced structures (Figure 3). We used the observed persistence length from TEM images as a measure of stiffness of the structures, as previously reported (Figure 3A).^31,32^ We designed and folded two structures: a vHelix hexagon rod (whose stiffness has been previously characterized^31,32^) and a reinforced rod with a square cross section. We used negative stained TEM pictures to estimate the persistence length. The estimated persistence length of the hexagon rod is 461 ± 68 nm, while for the reinforced rod it is 1730 ± 332 nm. The estimated persistence lengths of the two structures are in line with previous studies, and the difference between them confirms that the reinforced structures have a higher stiffness.

We also performed coarse-grained molecular dynamics simulations using the simulation software oxDNA to compare the rigidity of our reinforced structures with nonreinforced vHelix structures (Figure 3B).^20,38−40^ After the simulations, we used the suite of analysis tools for oxDNA to obtain the average configuration of the structures and estimate the root-mean-square fluctuation (RMSF) and the root-mean-square deviation (RMSD) during the simulations.^41,42^ The result on the icosahedral structure shows an increase in the rigidity of the single edges that can be appreciated by how the structure better maintains the designed shape and from the RMSF, which is higher for the nonreinforced structure than for the reinforced one (Figure 3B). The plotted RMSD also shows a much higher average RMSD for the nonreinforced structure than for the reinforced structure (6.3 ± 2.4 and 2.9 ± 0.7 nm, respectively), as expected. We also simulated the reinforced rod, in this instance by comparing it to both the nonreinforced version and to a vHelix hexagonal rod of similar diameter and length (Figure 3C). The hexagonal rod has been proved in previous studies to be one of the most rigid rod-type vHelix structures,^32^ so we used it as a well-characterized benchmark structure. The results are in line with the ones we obtained for the icosahedron: the reinforced structure shows a very good improvement in rigidity compared with the nonreinforced one, both qualitatively (the shape of the structure) and quantitatively (the average RMSF and RMSD of 6.3 ± 1, 3.2 ± 0.75, and 3 ± 0.6 nm for the nonreinforced rod, the reinforced rod, and the hexagon rod, respectively).

### Hybrid Structures of Tunable Stiffness

**Figure 4:**
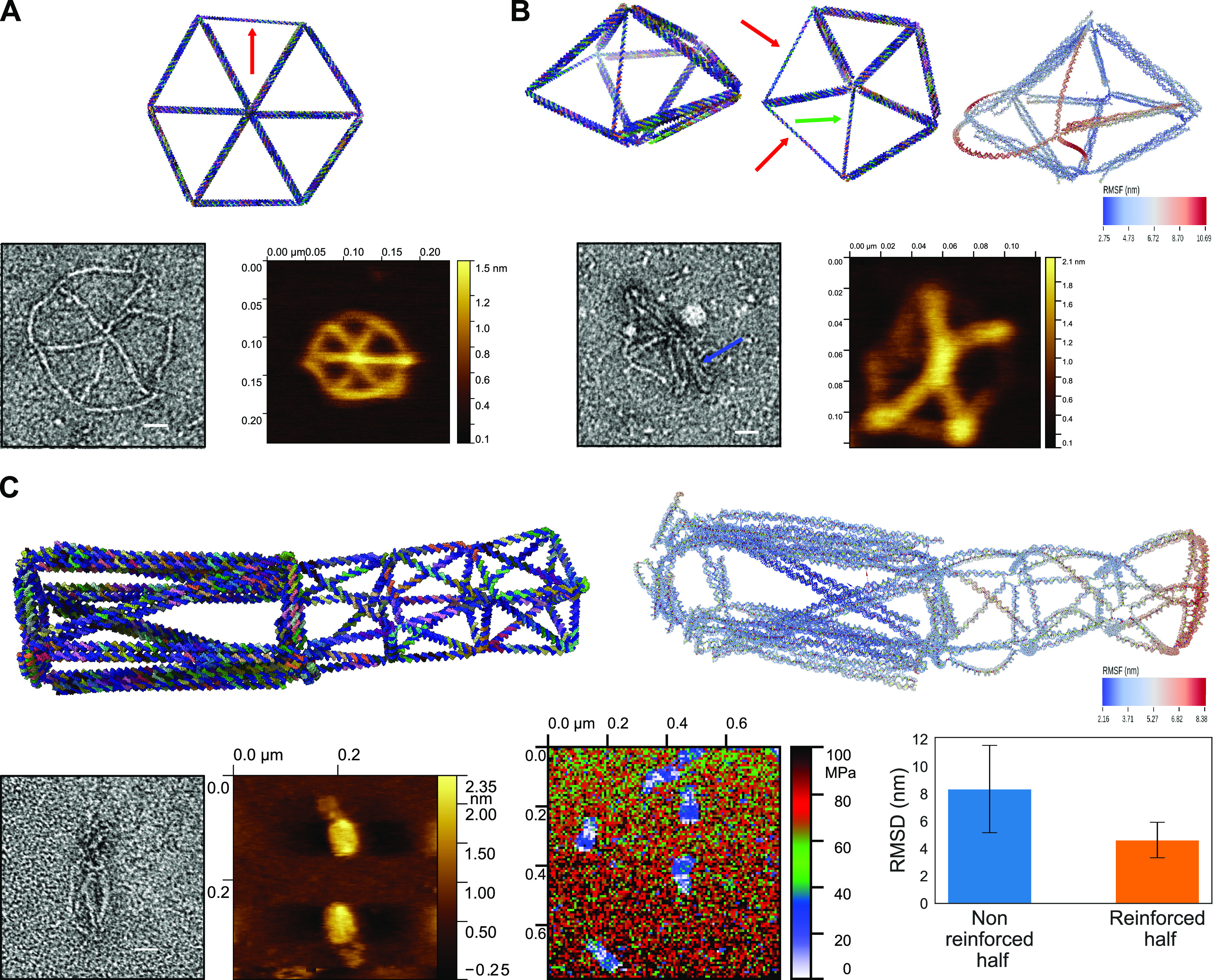
Validation and study of hybrid structures. (A) Hybrid hexagon. One edge nonreinforced. Top: rendering of the design; the red arrow indicates the nonreinforced edge. Bottom: negative staining TEM and AFM pictures. Scale bars are 20 nm. (B) Hybrid pentagonal bipyramid. Top: rendering of the design of the structure. Four edges are not reinforced (the red arrows indicate nonreinforced edges, the green arrow indicates overlapping nonreinforced edges), alternated with reinforced edges. Bottom: negative staining TEM and AFM images. The blue arrow indicates the reinforced edge included between nonreinforced edges. Scale bars are 20 nm. (C) Hybrid rod. Half of the structure is reinforced, and half is not. First row: left, rendering of the structure; right, result of oxDNA simulation. Bottom row: left, negative staining TEM and AFM characterization of the structure; center, apparent elastic modulus of the structures; right, plotted RMSD of the two halves of the structure. Scale bars are 20 nm.

We next proceeded to design what we call hybrid structures to demonstrate the flexibility of our design method. These structures consist of some reinforced edges, while others are not reinforced. To investigate the characteristics of this type of wireframe structures, we designed three structures: (i) a flat hexagon mesh, bearing all except one reinforced edge; (ii) a pentagonal bipyramid, where approximately half of the edges are reinforced; and (iii) a rodlike structure, with half of the edges reinforced (Figure 4).

We folded the hexagon mesh and analyzed it using negative staining TEM and atomic force microscopy (AFM) (Figure 4 A). In the TEM pictures, it is possible to see that five edges of the structure are easily recognizable, while one of them seems to be either not present or too small to observe. It seems that the structure is properly folded, but the flexibility of the nonreinforced edge makes it hard to fully confirm this under negative staining conditions. The AFM pictures do confirm correct folding and show structures with a single, thinner, nonreinforced edge (made only of dsDNA).

The hybrid pentagonal bipyramid allowed us to study the characteristics of 3D structures with multiple, longer nonreinforced edges interspaced between reinforced edges (Figure 4 B). The EM pictures show that the reinforced edges of the structure are folded, but the nonreinforced edges are barely detectable. From the AFM data, it is possible to distinguish the reinforced edges, which are thicker and more linear, and the nonreinforced edges, which are thinner and more curved. From the oxDNA simulation, we can see how the reinforced part of the structure is more rigid, while the nonreinforced part has a lower rigidity. In particular, one of the reinforced edges also shows significant fluctuations, probably because it is connected to three nonreinforced edges; this low rigidity is probably the cause of the difficulties in imaging the nonreinforced part of the structure under electron microscopy.

The hybrid rod was folded and analyzed in a similar way (Figure 4 C). Under EM, the reinforced edges are clearly distinguishable, and the length of the reinforced part is similar to the designed one, while the nonreinforced parts are harder to distinguish, and their lengths seem lower than expected. To determine whether the nonreinforced part was properly folded, we performed AFM in liquid and analyzed the pictures. In these pictures, it is possible to see that the nonreinforced part of the structures is also folded: the reinforced part appears larger than the nonreinforced one. We also used the AFM in liquid, under quantitative imaging (QI) mode, to explore the difference in stiffness between the reinforced and the nonreinforced parts of the hybrid rod.^31,43^ We mapped the apparent elastic modules of the structures when under a load of 164 pN (Figure 4 C). It is possible to see how the enlarged areas of the reinforced half of the structures is associated with a higher apparent Young’s modulus than the nonreinforced parts. This measurement indicates that there is a difference in the stiffness between the reinforced and nonreinforced parts of the hybrid rod. Considering the expected deformation due to the mica surface, though, we acknowledge that this result is mostly qualitative. We also simulated the structure using oxDNA to corroborate our experimental data on the difference of stiffness in the structure. The simulation shows a gradient in the RMSF of the structure where the reinforced part has a lower RSMF, while the nonreinforced part has a higher RMSF that increases the further away it is from the reinforced part of the structure. This indicates a higher flexibility in the nonreinforced part than in the reinforced part, which is also indicated by a lower RMSD for the reinforced half of the structure.

## Conclusions

Here, we present a way to design wireframe DNA origami structures on the basis of A-trail scaffold routing where the use can freely define parts of the structure’s edges to carry multiple helices. We used four helix bundles on a square lattice, with three of the bundles branching out from the scaffold without changing the underlying routing and one on synthetic mini-scaffolds. We demonstrated the correct folding of the structures and characterized how this strategy can be used to increase the rigidity of these types of wireframe structures experimentally and computationally. In addition, we demonstrated a type of hybrid structure by combining reinforced and nonreinforced edges, thereby presenting different mechanical properties in the same structure. This is possible thanks to the A-trail routing of the scaffold, which allows the use of more flexible single-DNA helices as edges that can be combined with more rigid bundles of multiple helices. Using this strategy, a user can easily modify the mechanical stiffness of the structure. Local rigidity has been shown to be of importance for the interactions of DNA origami nanostructures with cells. We argue that by expanding the design repertoire of A-trail routed wireframe structures in this way, the structures could find use in applications such as mechanobiology, by creating dynamic wireframe nanomachines that interact with cell receptors,^44^ and nanomedicine, by designing drug delivery vehicles with tunable penetration behavior into tissues.^31^

## Methods

### Nanostructures Design

The vHelix structures were designed using the BSCOR-vHelix software.

The reinforced structures were designed using our script and GUI. The GUI that performs design of 2D and 3D nanostructures from the target mesh is available on GitHub (https://github.com/marlol4/4vHelix). Additional information on the software is available in the Supporting Information (Supplementary Note S1).

The structures have been submitted to Nanobase.org^45^ for easy viewing (accession numbers 193 and 194).

### DNA Nanostructures Assembly

DNA nanostructures were folded in a solution of 10 or 20 nM scaffold (p7560, produced as previously reported^46^), a 10× excess of staples and mini-scaffolds (Integrated DNA Technologies), 5 mM TRIS (VWR), 1 mM EDTA (VWR), and a certain concentration of MgCl_2_ (Sigma-Aldrich) that depended on the structure (Table S1). The folding conditions for the structures, including salt concentrations, temperature, and length of the folding programs, were optimized for each structure (Table S1). The folding reactions were performed in a Techtum Gene Explorer 48 Dual Block Thermal Cycler. The structures were initially checked by agarose gel electrophoresis. In a 0.5× TRIS/Borate/EDTA (TBE) buffer supplemented with 10 mM MgCl_2_ and 0.5 mg/mL of ethidium bromide were cast 2% agarose gels. The gels were run in 0.5× TBE buffer supplemented with 10 mM MgCl_2_ at 90 V for 3.5 h. To avoid overheating, the gels were run in an ice water bath. After they were run, the gels were imaged using a GE LAS 4000 imager. The folding yield was estimated from the intensity of the band on the agarose gel using ImageJ (Supplementary Tables S2 and S3). The excess staple strands were removed either by ultrafiltration or PEG-precipitation. For the ultrafiltration, 100  kDa cutoff filters were used (Amicon, Millipore). The structures were transferred to the tube and diluted to 500 μL with a buffer containing 10 mM MgCl_2_, 5 mM TRIS, and 1 mM EDTA. The sample was then centrifuged at 4000*g* for 2.5 min. This process was repeated five times. The PEG precipitation was performed as previously reported.^46^

### Atomic Force Microscopy

For the imaging in air of the structures (hybrid hexagon and hybrid pentagonal bipyramid), the purified structure was diluted to 0.5 nM in a buffer containing 10 mM MgCl_2_, 5 mM TRIS, and 1 mM EDTA. Ten μL of structure were then added onto a freshly cleaved mica surface and incubated for 1 min. The surface was then washed five times with water and blow dried. The imaging was conducted with a JPK instruments Nanowizard 3 ULTRA in AC mode using ScanAsyst-Air (Bruker) cantilever with a nominal spring constant of 0.4 N/m. The resulting data was processed using Gwyddion. For the imaging in liquid, 10 μL of purified and diluted structure were added to a freshly cleaved mica surface and incubated for 30 s. A 5 μL aliquot of NiSO_4_ was added for a further 4.5 min incubation. The surface of the sample was then washed with 1 mL of imaging buffer (10 mM MgCl_2_, 5 mM TRIS, and 1 mM EDTA), and then the sample was imaged in 1.5 mL of imaging buffer. The imaging was conducted with a JPK instruments Nanowizard 3 ULTRA in AC mode using an AC40 (Bruker) cantilever with a nominal spring constant of 0.09 N/m. The resulting data was processed using Gwyddion. For the analysis of the mechanical properties of the DNA origami structures, we used JPK instruments Nanowizard 3 ULTRA in quantitative imaging (QI) mode. The measurement was performed in liquid. We used an AC40 (Bruker) cantilever with a nominal spring constant of 0.09 N/m. In QI, we obtained force-identification profiles by positioning the AFM probe on top of the sample and pressing at a velocity of 5 μm/s with a set point of 0.164 nM. The raw data was processed using JPK Data Processing Software and by applying a Hertz model to the curves. The data were then further processed using Gwyddion.

### Negative Staining TEM

A droplet of 3.5 μL of 5 nM structure sample was spotted on a glow-discharged, carbon-coated, Formvar resign grid (Electron Microscopy Sciences) for 20 s before blotting on filter paper. The grid was then stained with 2% w/v aqueous uranyl formate solution. The stained sample was imaged using a Talos 120C transmission electron microscope at 120 kV.

### Edge Length Analysis from TEM Data

For each pentagonal structure, we picked enough structure from the negative staining EM images to guarantee to have at least 100 properly folded edges. The vertex-to-vertex distance was measured manually from the single structures using ImageJ and plotted using custom Python scripts.

### Persistence Length Estimation from TEM Data

The persistence length was estimated as previously reported.^31,32^ For each sample, we collected TEM images of at least 100 structures. The persistence length was calculated using a custom Python script. For each structure, a spine was created through it by positioning points 11 nm apart along the structure. The correlation between tangent vectors separated by a distance *l* along the trajectory of a structure is expected to decay according to the equation:
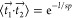
1where *p* is the persistence length, and *s* is a surface parameter set to 2 for structures that have equilibrated on a surface.^47^ The software creates tangent vectors between adjacent points along the spine of the structures and then calculates cosines of the angles between vector pairs of increasing distance along the spine. To this data, eq 1 is fitted to estimate the persistence length *p*.

### Coarse-Grained Simulation of DNA Nanostructures

The structures for simulations were converted to oxDNA format using the Web server tacoxDNA.^48^ The output from tacoxDNA was then loaded in the Web server oxView and relaxed using the rigid-body simulation tool.^41,42^ After this step, the structures were relaxed in two steps. The first step is a minimization step run for 2 × 10^5^ steps to remove possible overlapping nucleotides. The second step is a molecular dynamics simulation run for 5 × 10^6^ steps, with a maximum backbone force of 50, to reduce overstretched bonds. After these two relaxation steps, the structures were simulated for 1 × 10^8^ steps, with a time step of 0.005 oxDNA time units. The simulations were performed with the oxDNA2 model at 30 °C with a salt concentration of 0.15 M and an Anderson-like thermostat. The simulation states were saved every 2 × 10^4^ steps. After simulation, the average structure, root-mean-square fluctuation (RMSF), and root-mean-square deviation (RMSD) were calculated using the oxDNA analysis tools.^41,42^

## Supporting Information Available

Supplementary Note S1; comparison between spanning-tree routing and A-trail routing of scaffolds (Supplementary Figure S1); TEM collages of structures (Supplementary Figures S2–S8); additional TEM pictures of structures (Supplementary Figures S9–S16); AGE of the structures presented in this work (Supplementary Figures S17–S22); raw RMSD plots from the oxDNA analysis tool (Supplementary Figures S23–25); caDNAno designs of the structures presented in this work (Supplementary Figures S26–S38); folding conditions for the structures presented in this work (Supplementary Table 1); and estimations of folding yields for the structures presented in this work (Supplementary Tables 2–3) (PDF)
