# Supplementary material for: Computer-Aided Design of A-Trail Routed Wireframe DNA Nanostructures with Square Lattice Edges
Source: ACS Nano. 2023 Mar 23;17(7):6565–74. doi: 10.1021/acsnano.2c11982 (PMC10100577; doi:10.1021/acsnano.2c11982)
Supplement: Supplementary file 1 — nn2c11982_si_001.pdf [file nn2c11982_si_001.pdf]

Supporting information

Computer-aided design of A-trail routed wireframe DNA nanostructures with square lattice edges

Marco Lolaico[a], Sebbe Blokhuizen[a], Boxuan Shen[a,b], Yang Wang[a] and Björn Högberg[a]*

Affiliations:

a Department of Medical Biochemistry and Biophysics, Karolinska Institutet, SE-17177 Stockholm, Sweden

b Biohybrid Materials, Department of Bioproducts and Biosystems, Aalto University School of Chemical Engineering, P.O. Box 16100, 00076, Aalto, Finland

Supplementary note 1: Nanostructures' design

The input for the nanostructures' design is a PLY mesh file, representing a 2D or 3D mesh. In this file type, the mesh is represented as a list of vertices and faces. The scaffold routing through the target mesh is found using the package BSCOR available from www.vhelix.net [1,2]. The BSCOR package has different outputs, and two of them are needed for the reinforcement of the edges of the wireframe structures: the NTRAIL file, representing the sequence of vertices visited by the scaffold, and the final RPOLY file, representing the DNA helices after the relaxation has been completed in BSCOR.

The next steps are performed by the software developed for this work. After the edges to be reinforced are selected, from the RPOLY file the "main" helix of each edge to be reinforced is extracted and 3 other helices are created in the normal edges and 2 are created in the double edges (the NTRAIL file is necessary to find where the double edges are). Two of these helices are then connected to the main helix with crossovers, going to form a scaffold loop; the third one, if present, is left not connected and it will represent the mini-scaffold for that edge. The loops are shortened on the side, generally at least 10 bp on each side, to avoid collisions with other bundles at the vertices. In case of non-reinforced edges, only one helix of the scaffold is created.

Once the scaffold bundles are created, the staples are added. On the extremities of the bundle, the staple connections are dictated by the RPOLY scaffold routing; in the rest of the bundle, the algorithm finds all the possible staple crossovers that are further away than 5 bp from a scaffold crossover. Staple breaks are then introduced when the single staple reaches 32 bp in length, with one 16 bp "seed" [3]. Once the staples

are added to the scaffold, the bundles are finished. The next step is to connect the bundles to each other: this is done simply following the scaffold routing from the RPOLY file. The RPOLY file is also the base for the connections for the staples. To account for possible imprecisions in the relaxation procedure from BSCOR, we add a few bases to fill the gaps between helices. The bundles are modelled in 3D and the software calculates the distance between the helices. If there is a spacing, is then filled with bases (poly Ts in case of staple strands). Deletions are introduced every 48 bp [4] when the edge longer than 50 bp.

The output of the software is a JSON caDNAno file that contains the design of the structure, and 2 CSV files containing the sequences for the staples and the mini-scaffolds. The sequences in the mini scaffold are generated randomly by a custom Python script, following a few rules: having a GC content similar to the m13-based scaffold we are using (p7560) of around 42% [5] and no more than 44% and not having more than 4 Cs or Gs in a row, because it could make the synthesis more challenging.

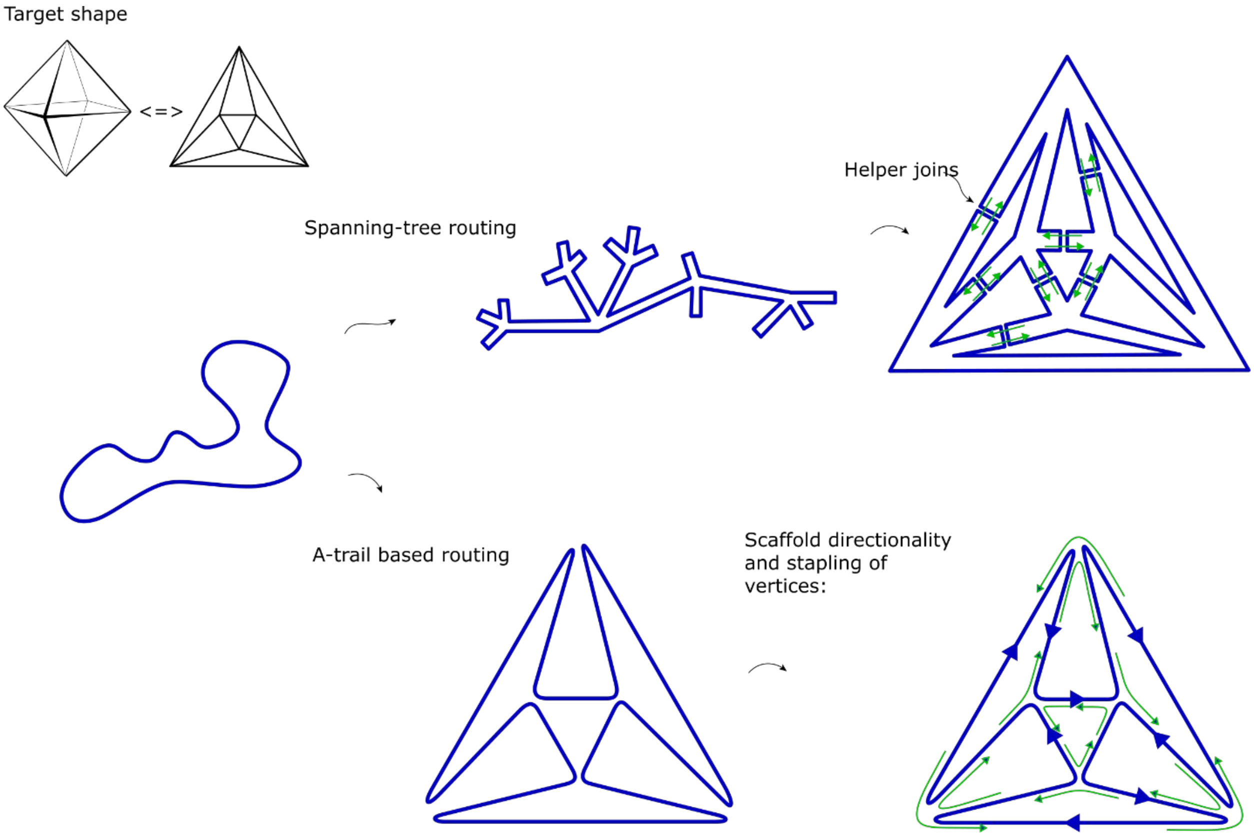

Supplementary Figure S1. Comparison between spanning tree routing and A-trail based routing. In blue is indicated the scaffold and in green the staples. On top, the target shape and its representation as a flattened Schlegel diagram. In the spanning-tree routing, the routing through the target shape is based on a tree-like representation, where each branch is composed of an even number of helices (in this case two) and the branches are connected using helper joins. In the A-trail routing, the scaffold route through the edges of the mesh, without crossing and preferably only traversing each edge once. In odd-degree vertices, this is not possible, and some edges must be traversed twice. This allows the creation of routing with mostly one helix per edge, and occasionally two helices per edge.

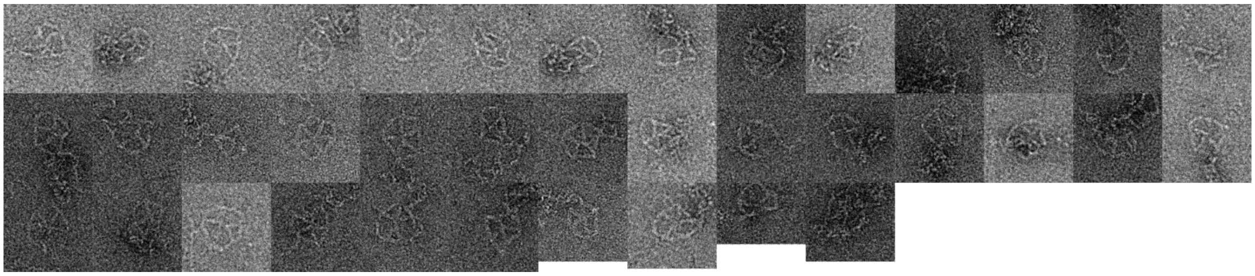

Supplementary Figure S2. TEM images of pentagonal mesh with circa 80 bp long edges.

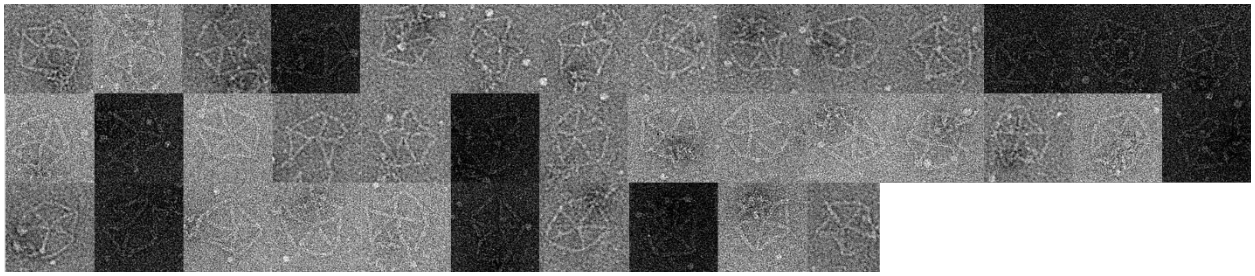

Supplementary Figure S3. TEM images of pentagonal mesh with circa 130 bp long edges.

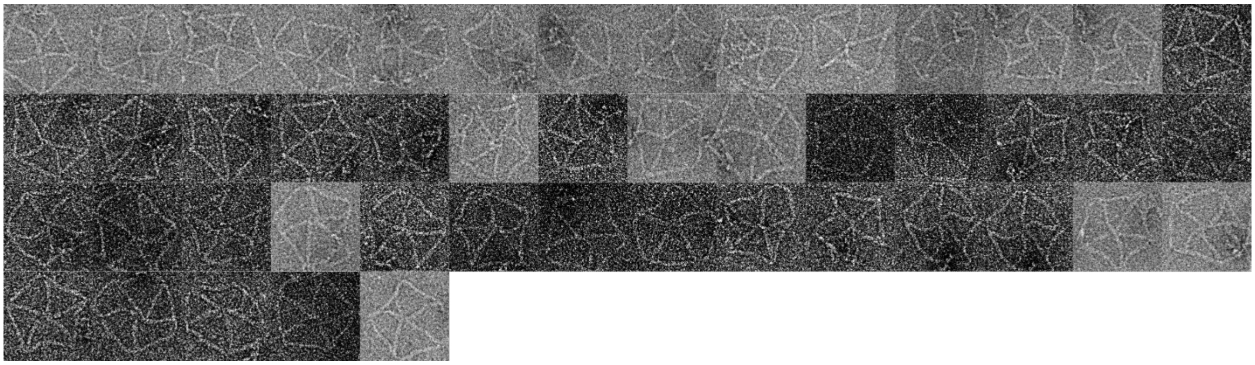

Supplementary Figure S4. TEM images of pentagonal mesh with circa 170 bp long edges

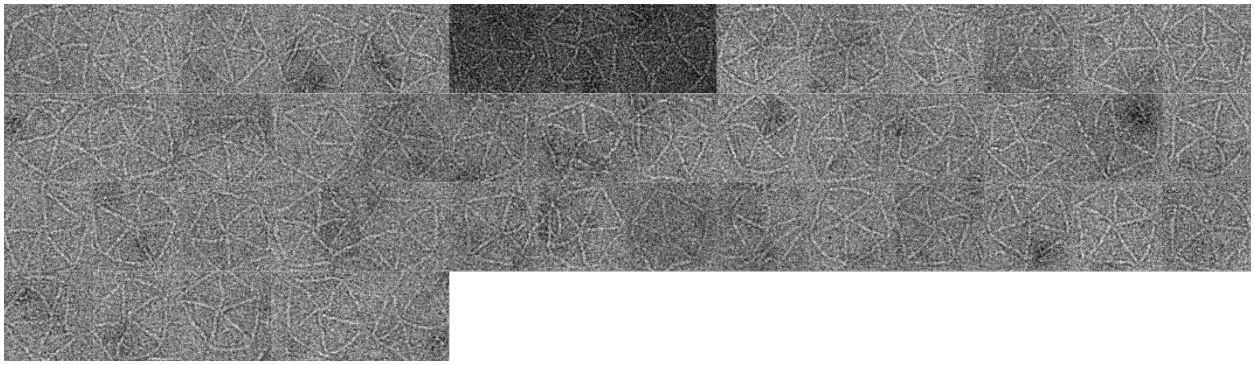

Supplementary Figure S5. TEM images of pentagonal mesh with circa 200 bp long edges

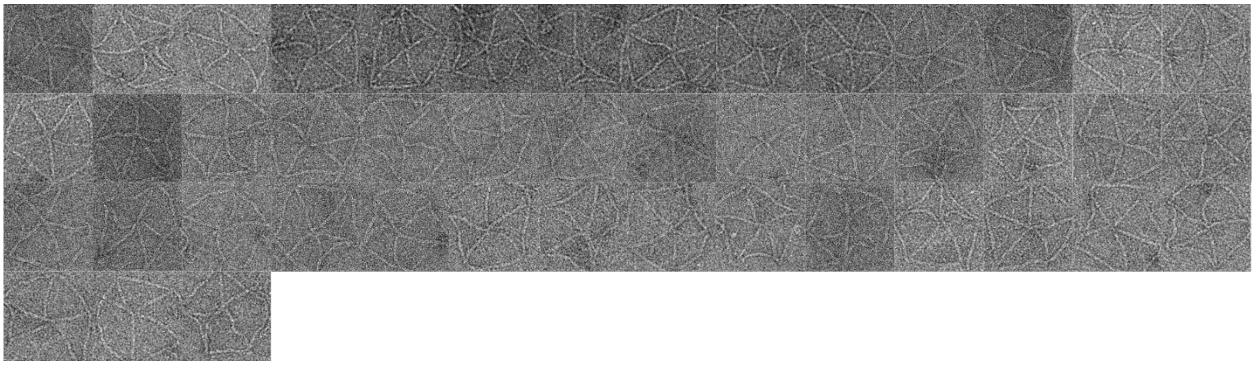

Supplementary Figure S6. TEM images of pentagonal mesh with circa 230 bp long edges

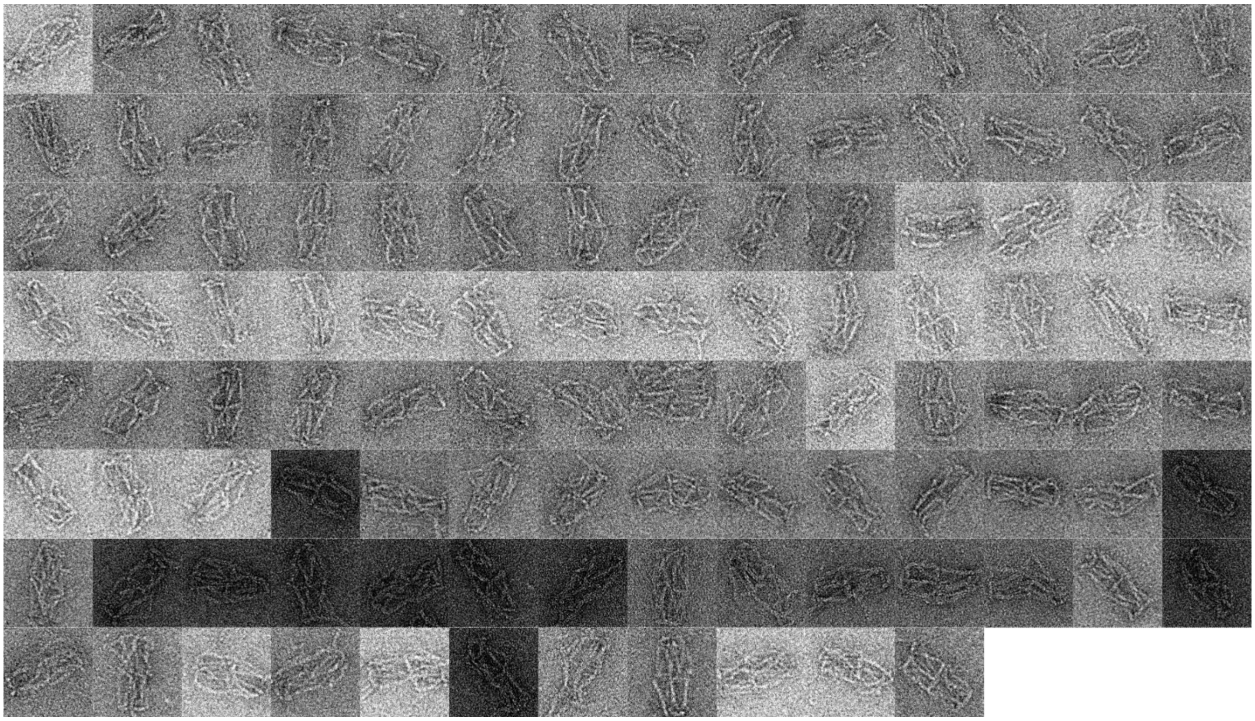

Supplementary Figure S7. TEM images of the reinforced rod used for the estimation of persistence length.

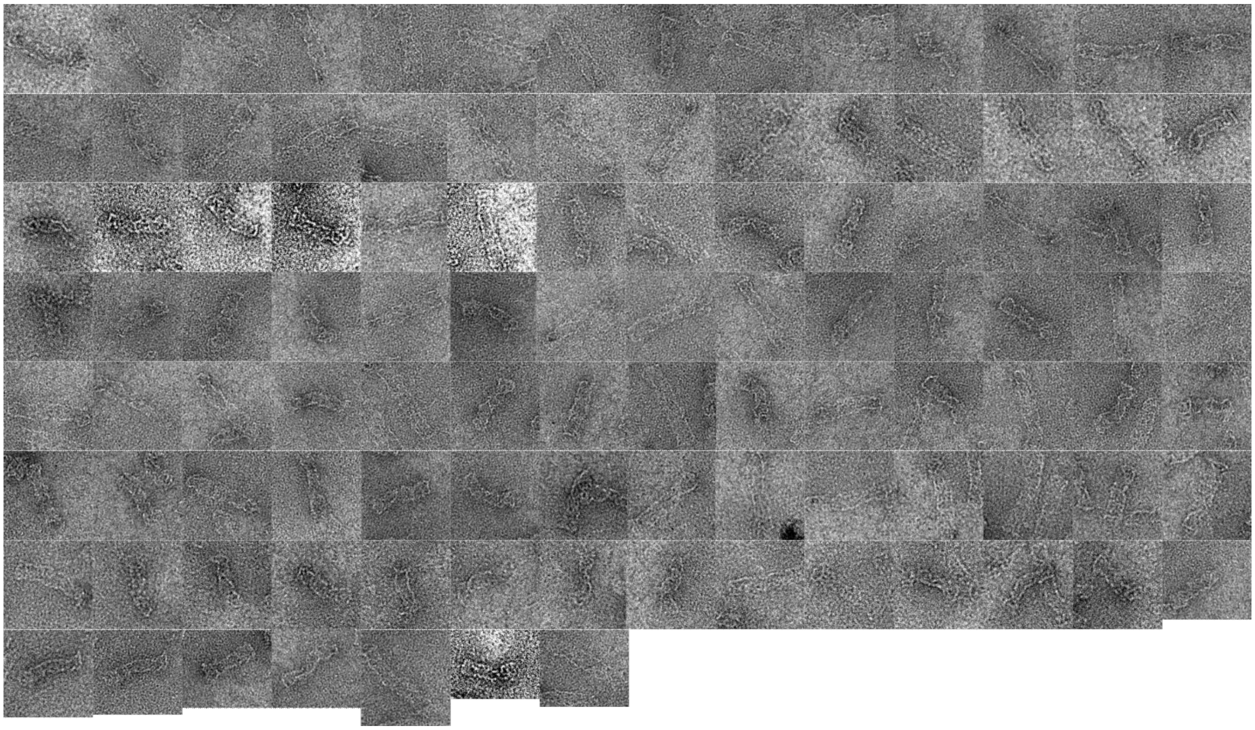

Supplementary Figure S8. TEM images of the vHelix hexagon rod used for the estimation of persistence length.

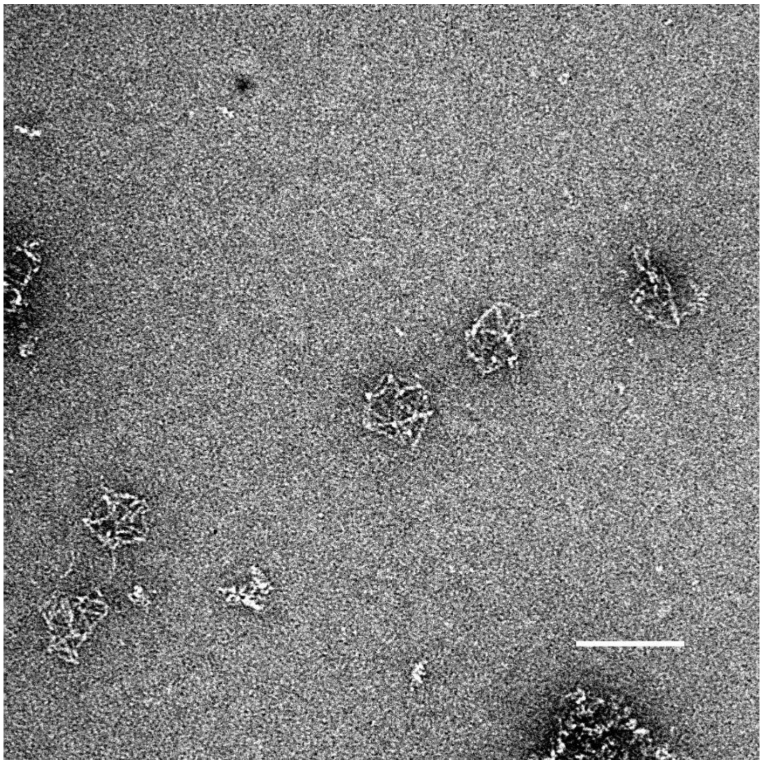

Supplementary Figure S9. TEM image of icosahedron. Scale bar 100 nm.

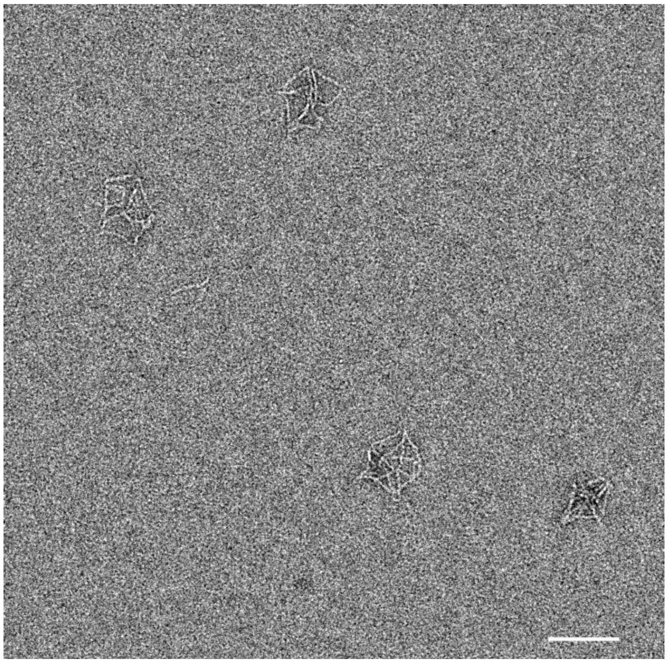

Supplementary Figure S10. TEM image of pentagonal bipyramid. Scale bar is 100 nm.

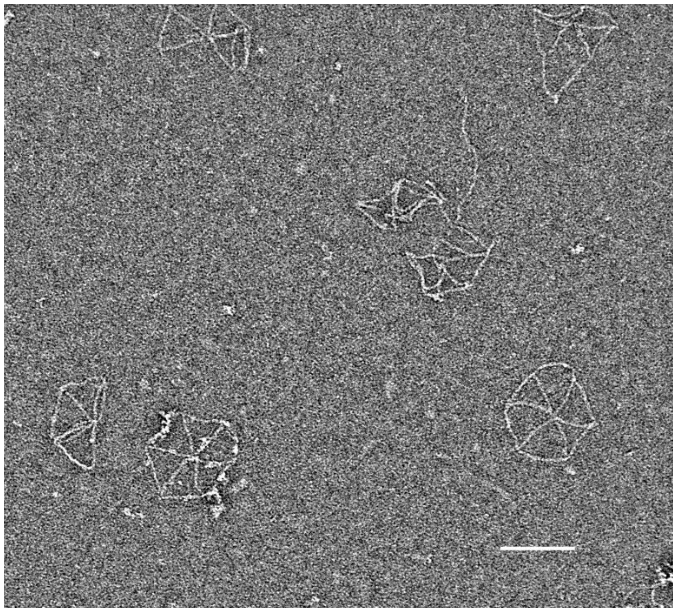

Supplementary Figure S11. TEM image of hexagonal mesh. Scale bar 100 nm.

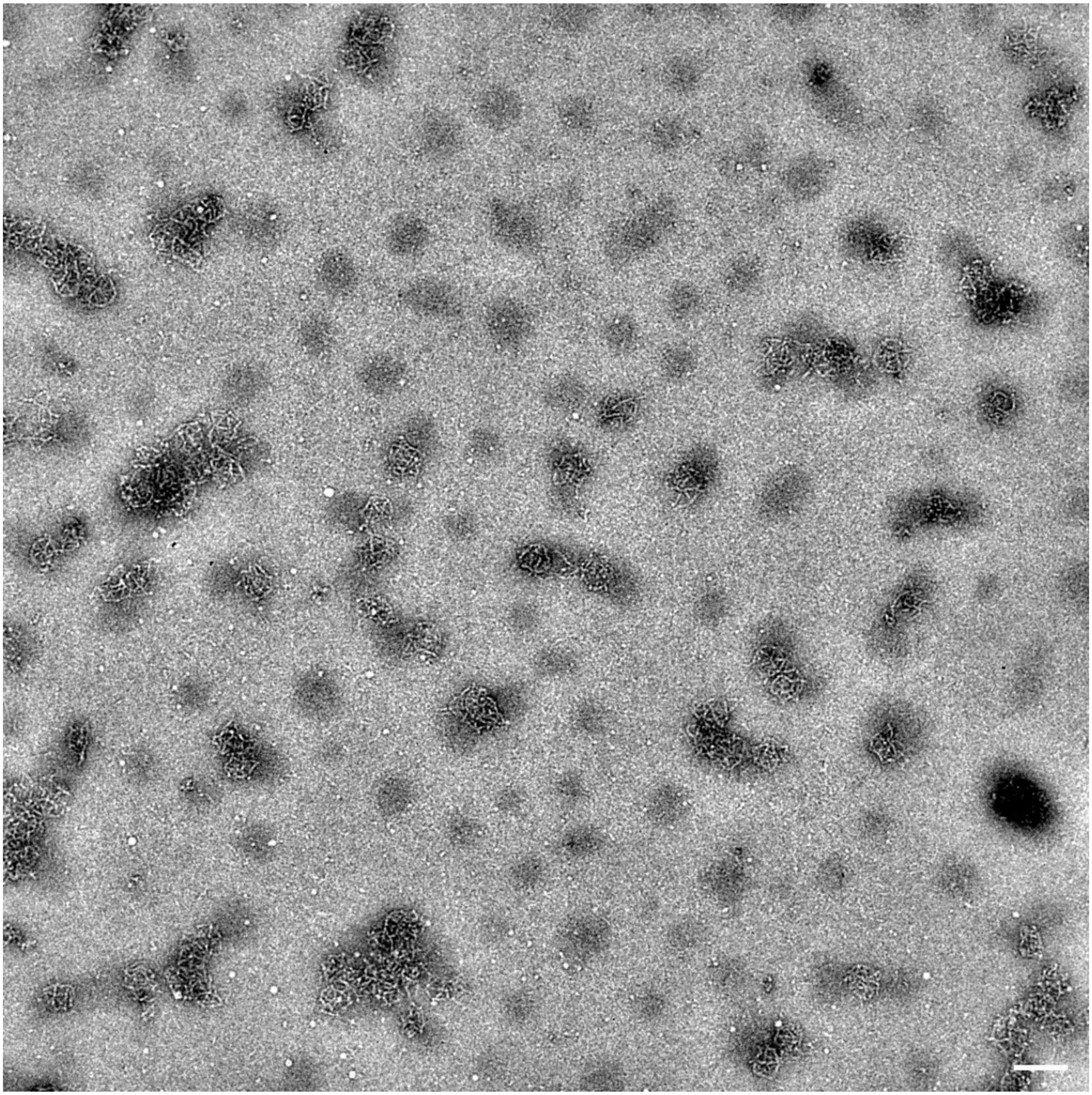

Supplementary Figure S12. TEM image of icosahedron. Scale bar 100 nm.

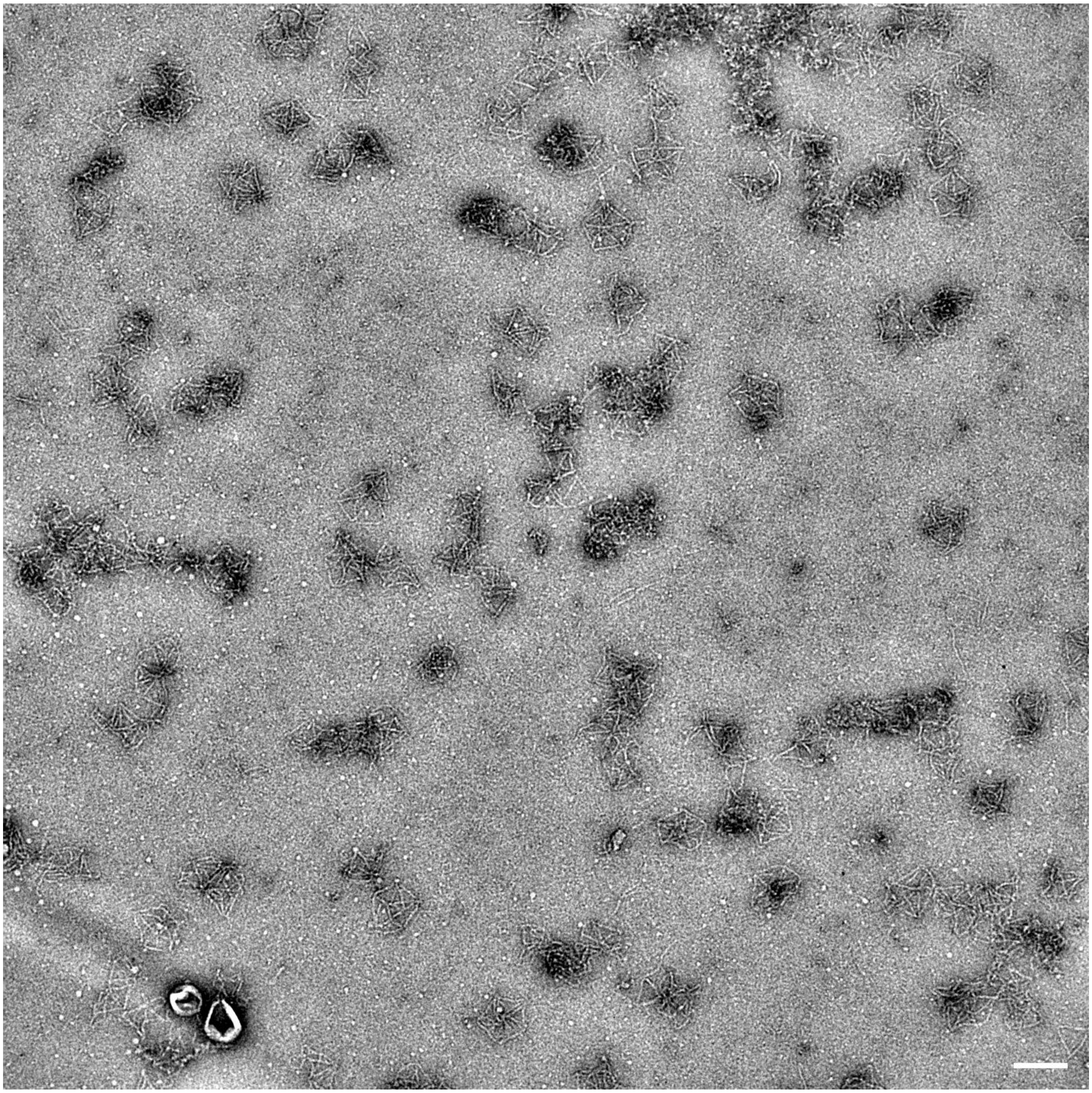

Supplementary Figure S13. TEM image of pentagonal bipyramid. Scale bar 100 nm.

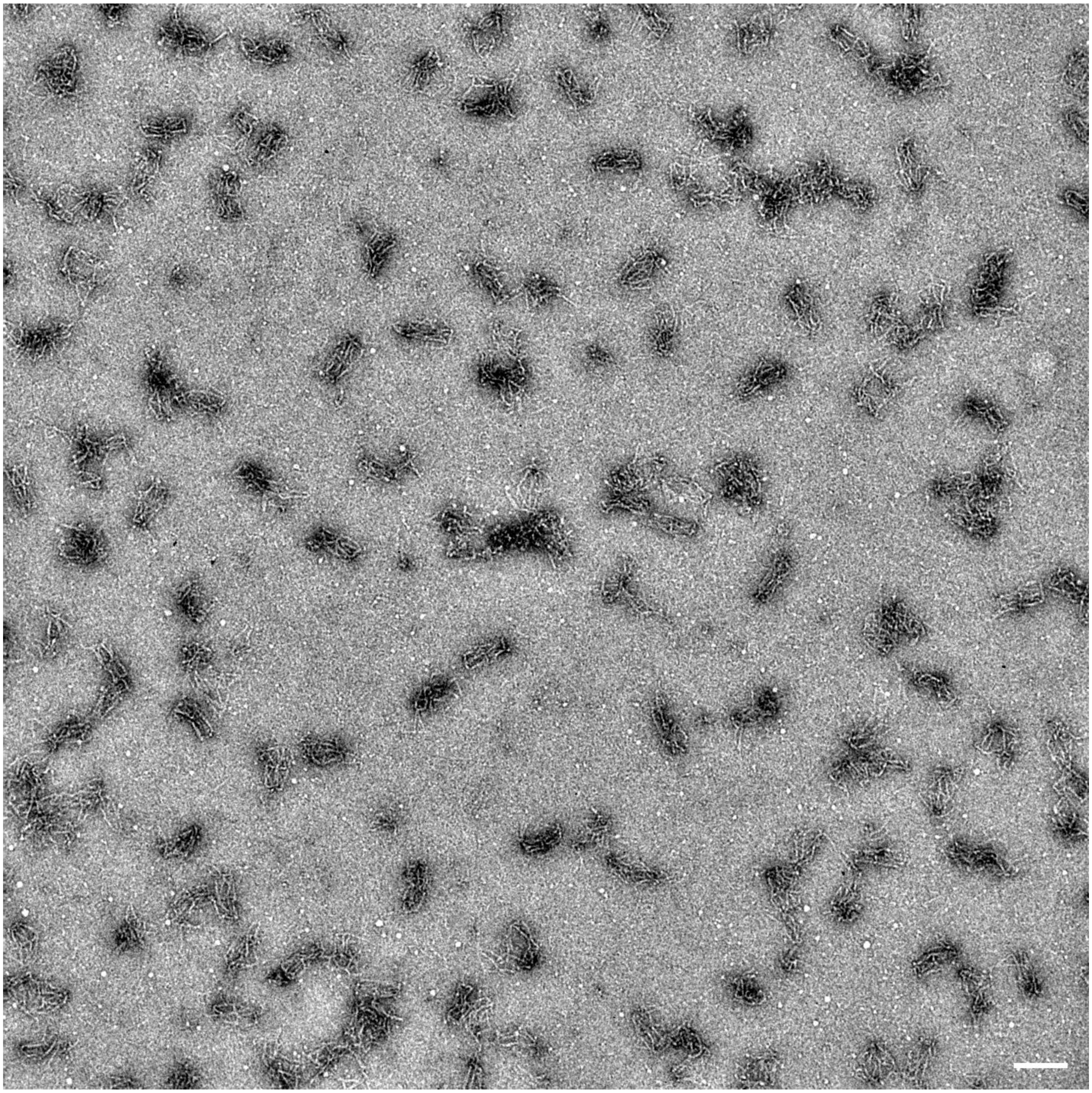

Supplementary Figure S14. TEM image of reinforced rod. Scale bar 100 nm.

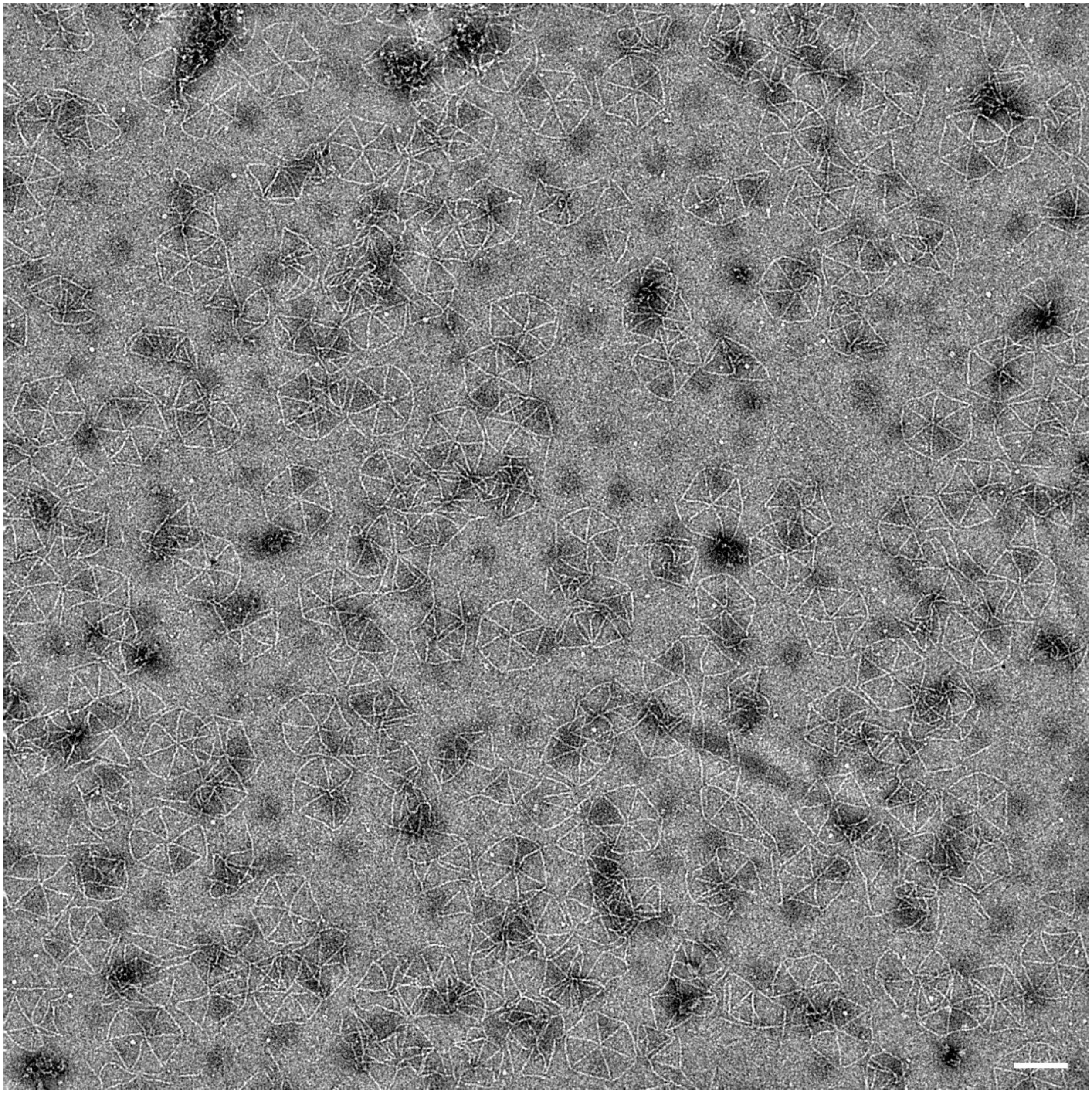

Supplementary Figure S15. TEM image of hexagon mesh. Scale bar 100 nm.

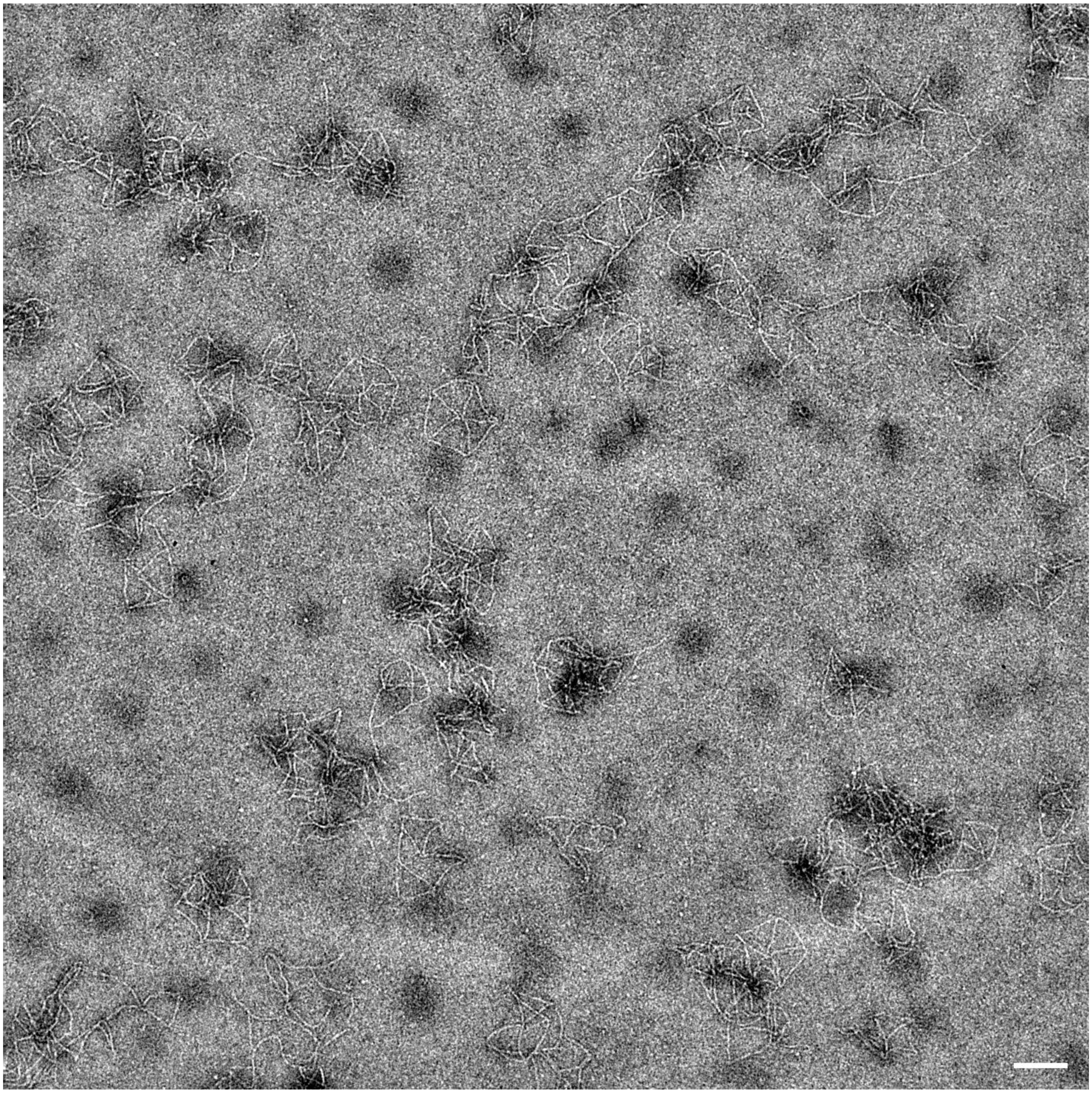

Supplementary Figure S16. TEM image of pentagon mesh. Scale bar 100 nm.

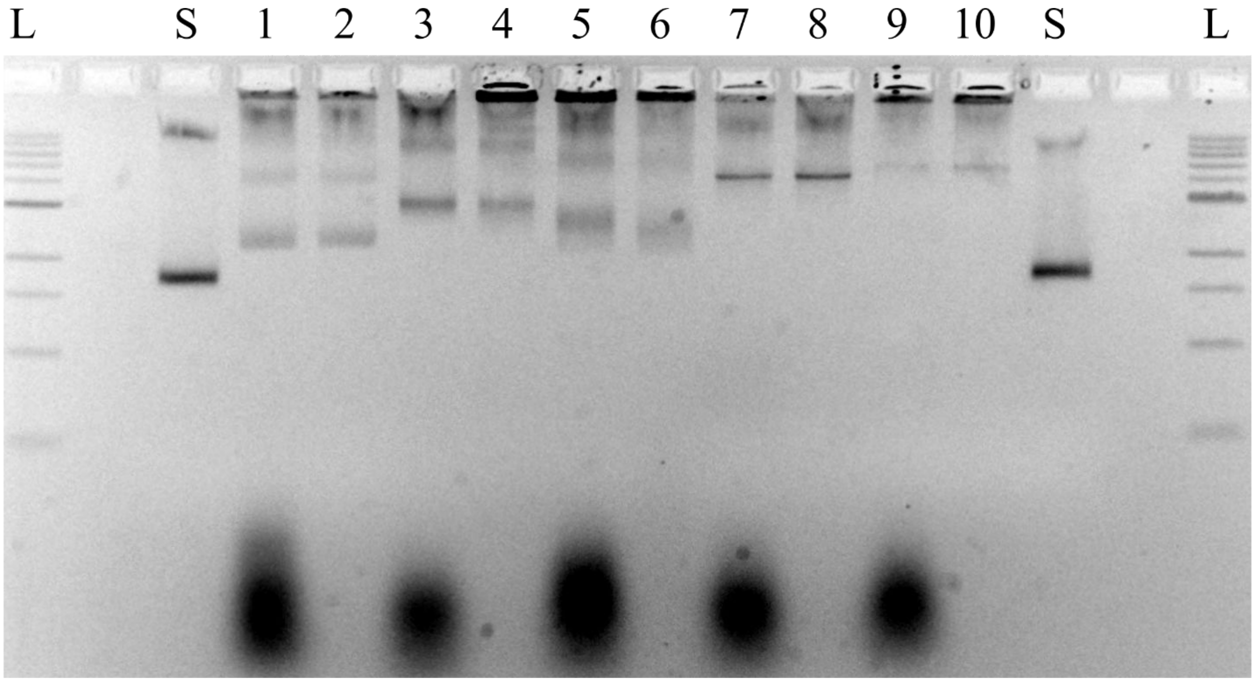

Supplementary Figure S17. 2% AGE of the reinforced structures presented in Figure 2. Lanes: 1. Icosahedron non purified. 2. Icosahedron purified 3. Pentagonal bipyramid non purified 4. Pentagonal bipyramid purified 5. Reinforced Rod non purified 6. Reinforced Rod purified 7. Hexagonal mesh non purified 8. Hexagonal mesh purified 9. Pentagonal mesh non purified 10. Pentagonal mesh purified S. Scaffold p7560 L. 1kb ladder.

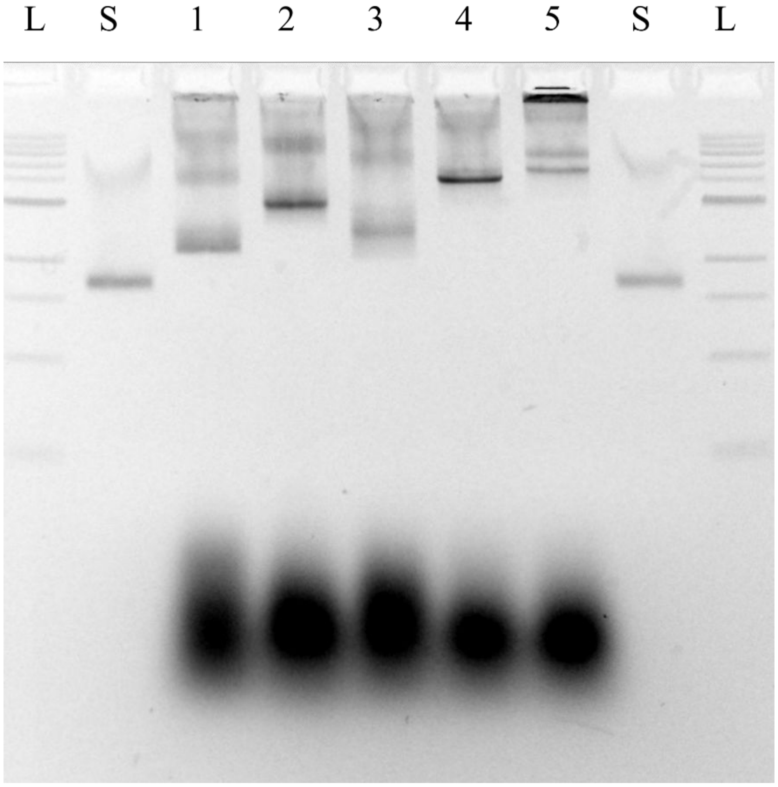

Supplementary Figure S18. 2% AGE of the reinforced structures presented in Figure 2, immediately after the folding with the 16 hours program. Lanes: 1. Icosahedron 2. Pentagonal bipyramid 3. Reinforced Rod 4. Hexagonal mesh 5. Pentagonal mesh S. Scaffold p7560 L. 1kb ladder.

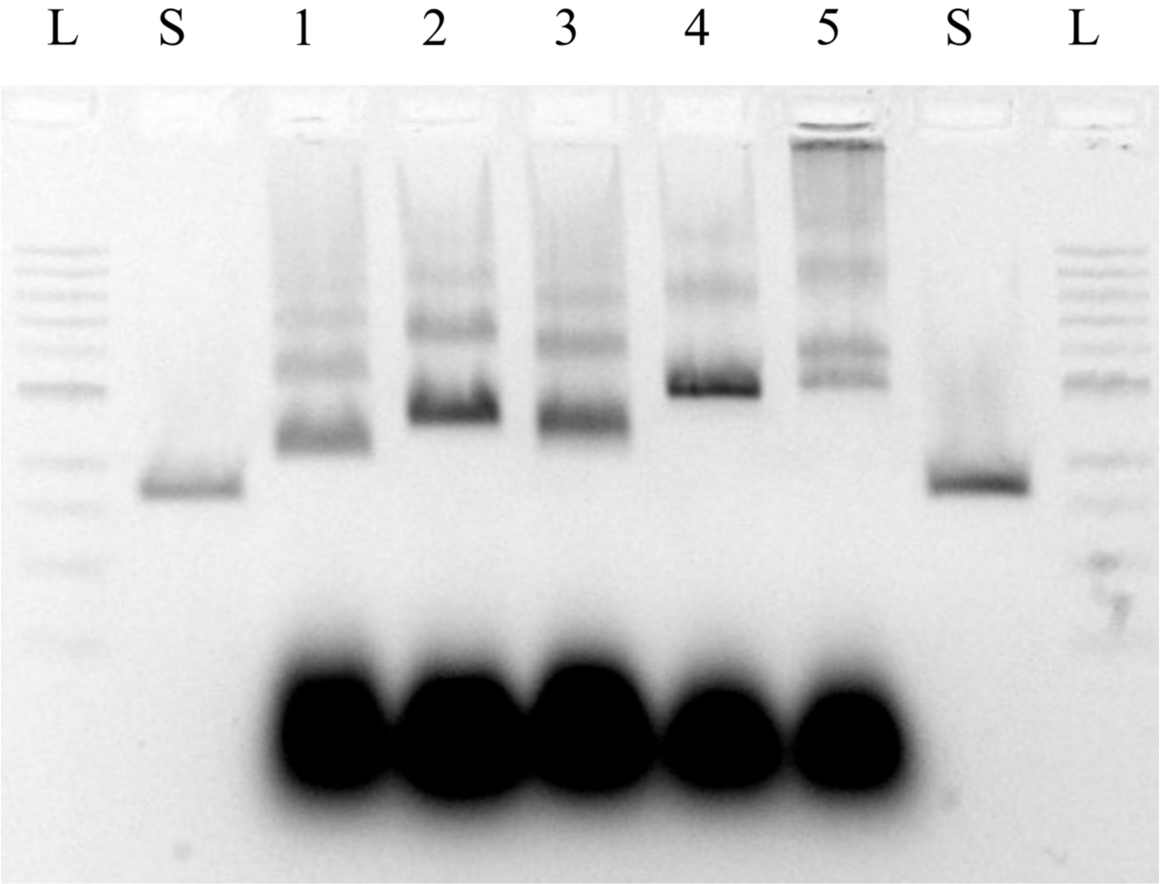

Supplementary Figure S19 1% AGE of the reinforced structures presented in Figure 2, immediately after the folding with the 16 hours program. Lanes: 1. Icosahedron 2. Pentagonal bipyramid 3. Reinforced Rod 4. Hexagonal mesh 5. Pentagonal mesh S. Scaffold p7560 L. 1kb ladder.

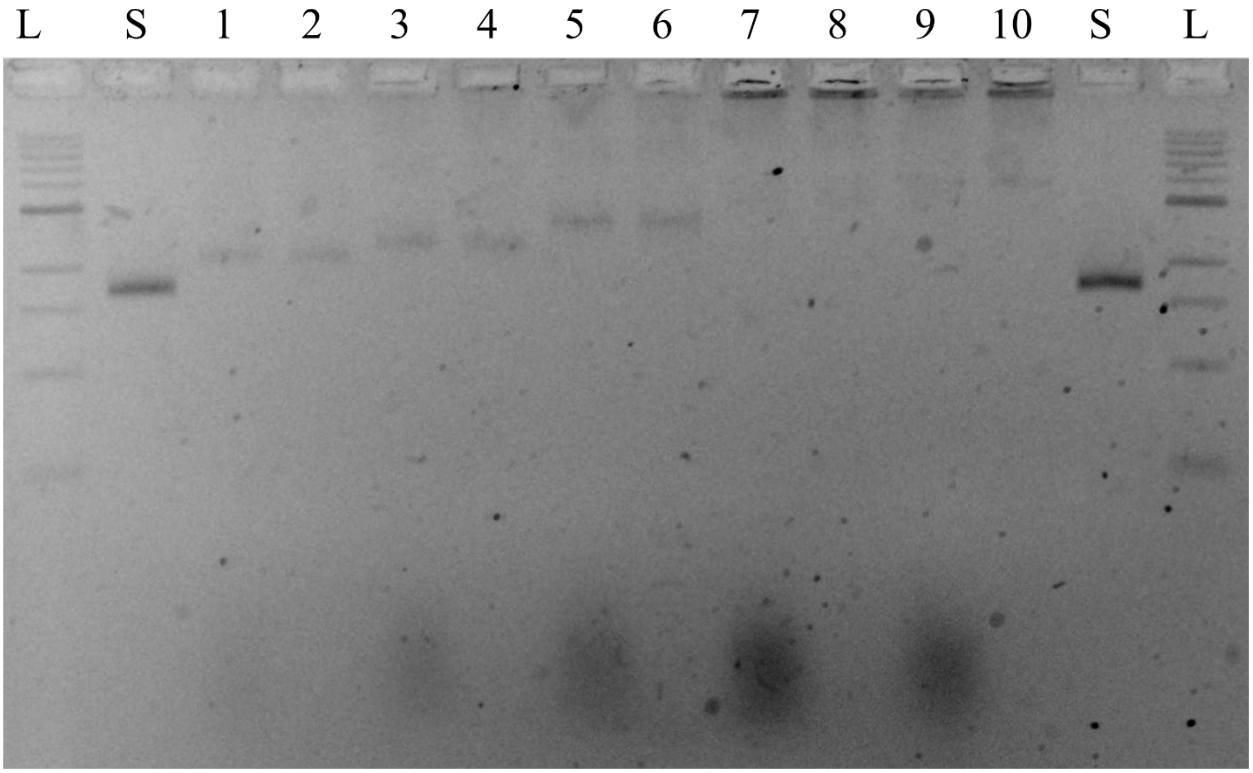

Supplementary Figure S20. 2% AGE of the different pentagonal meshes presented in Figure 2. Lanes: 1. Pentagon 80 bp edge non purified. 2. Pentagon 80 bp edge purified 3. Pentagon 130 bp edge non purified 4. Pentagon 130 bp edge purified 5. Pentagon 170 bp edge non purified 6. Pentagon 170 bp edge purified 7. Pentagon 200 bp edge non purified 8. Pentagon 200 bp edge purified 9 Pentagon 230 bp edge non purified 10. Pentagon 230 bp edge purified S. Scaffold p7560 L. 1kb ladder.

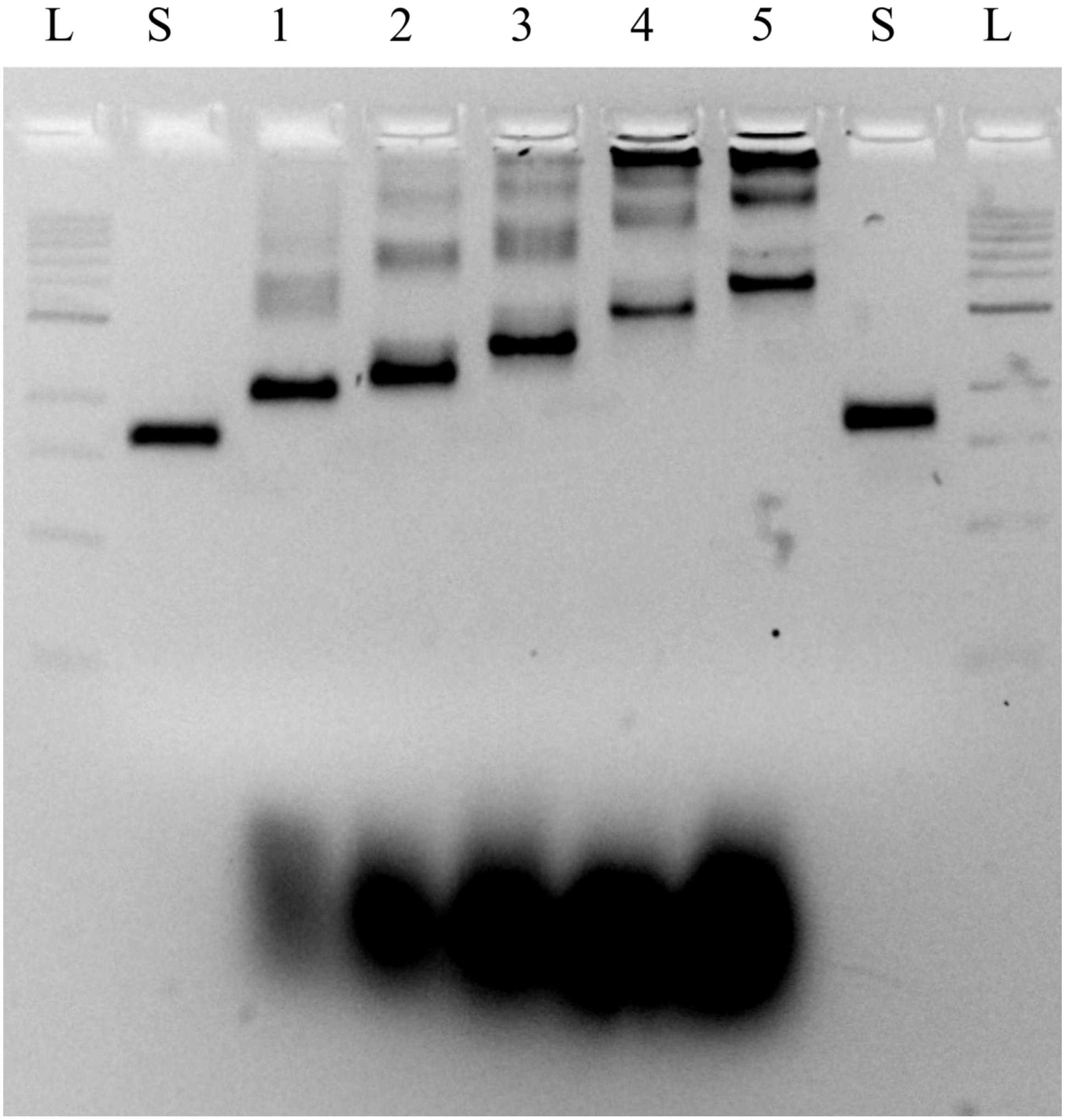

Supplementary Figure S21 2% AGE of the different pentagonal meshes presented in Figure 2, immediately after folding with the 16 hours program. Lanes: 1. Pentagon 80 bp edge 2. Pentagon 130 bp edge 3. Pentagon 170 bp edge 4. Pentagon 200 bp 5. Pentagon 230 bp S. Scaffold p7560 L. 1kb ladder.

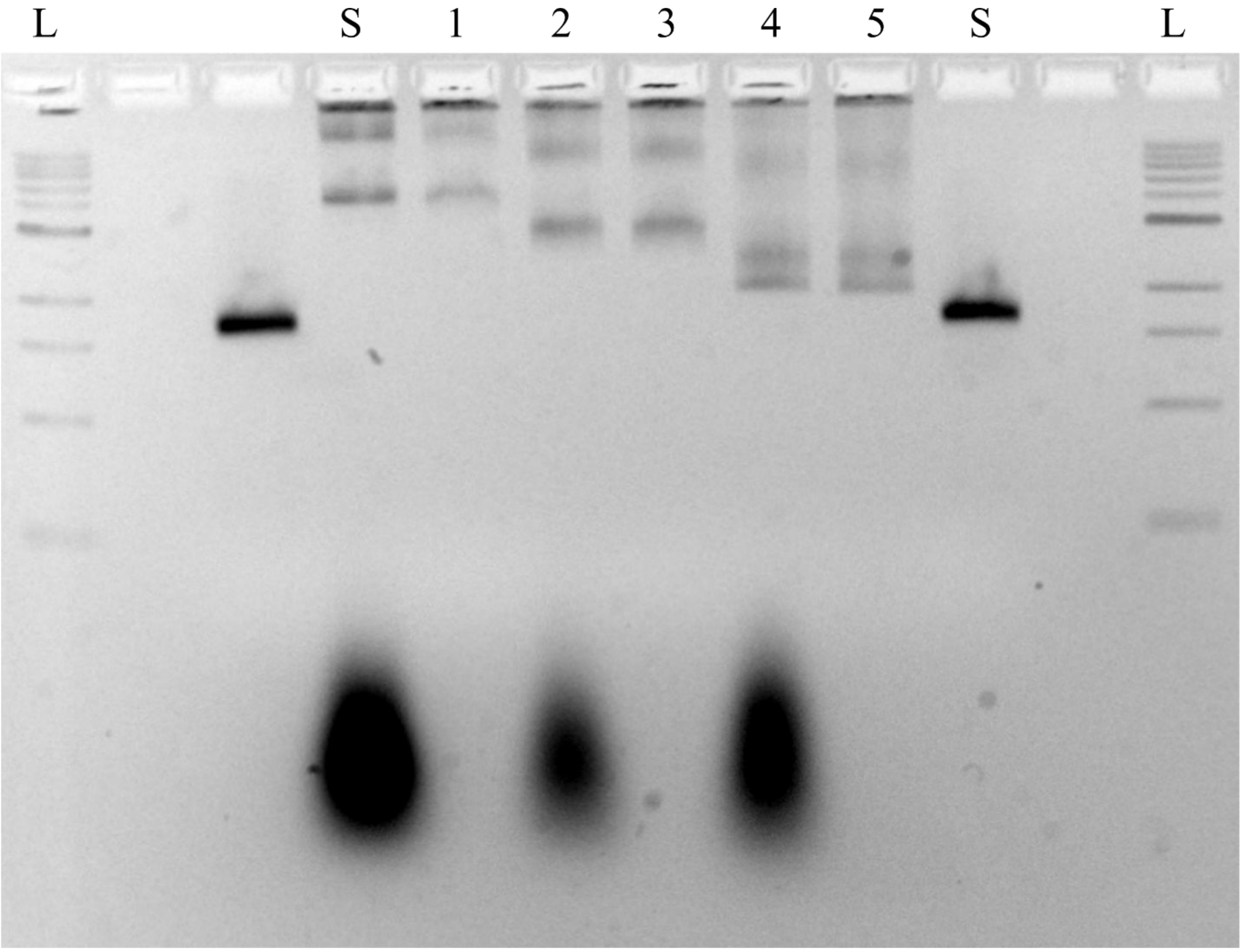

Supplementary Figure S22. AGE of hybrid structures presented in Figure 4. . Lanes: 1. Hybrid hexagon non purified. 2. Hybrid hexagon purified 3. Hybrid pentagonal bipyramid non purified 4. Hybrid pentagonal bipyramid purified 5. Hybrid rod non purified 6. Hybrid rod purified S. Scaffold p7560 L. 1kb ladder

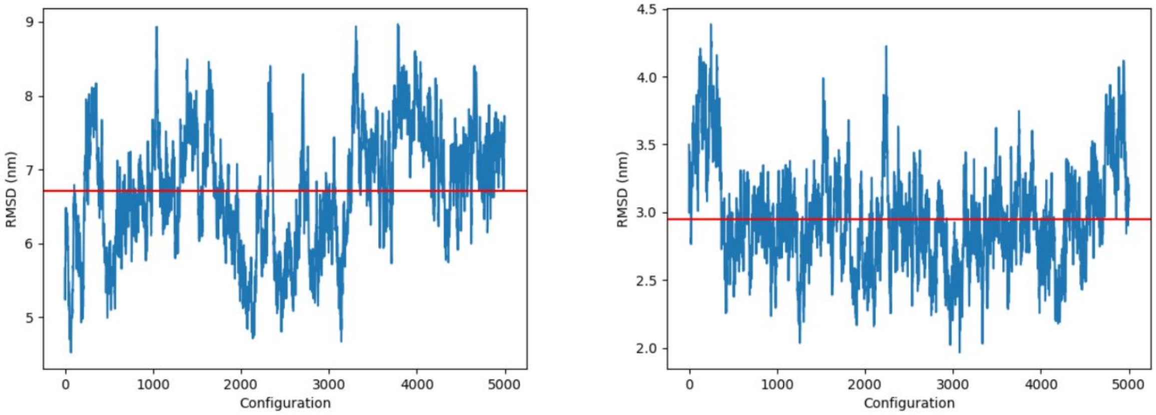

Supplementary Figure 23. Plot of the RMSD obtained from the oxDNA simulations for the non-reinforced icosahedron and for the reinforced icosahedron.

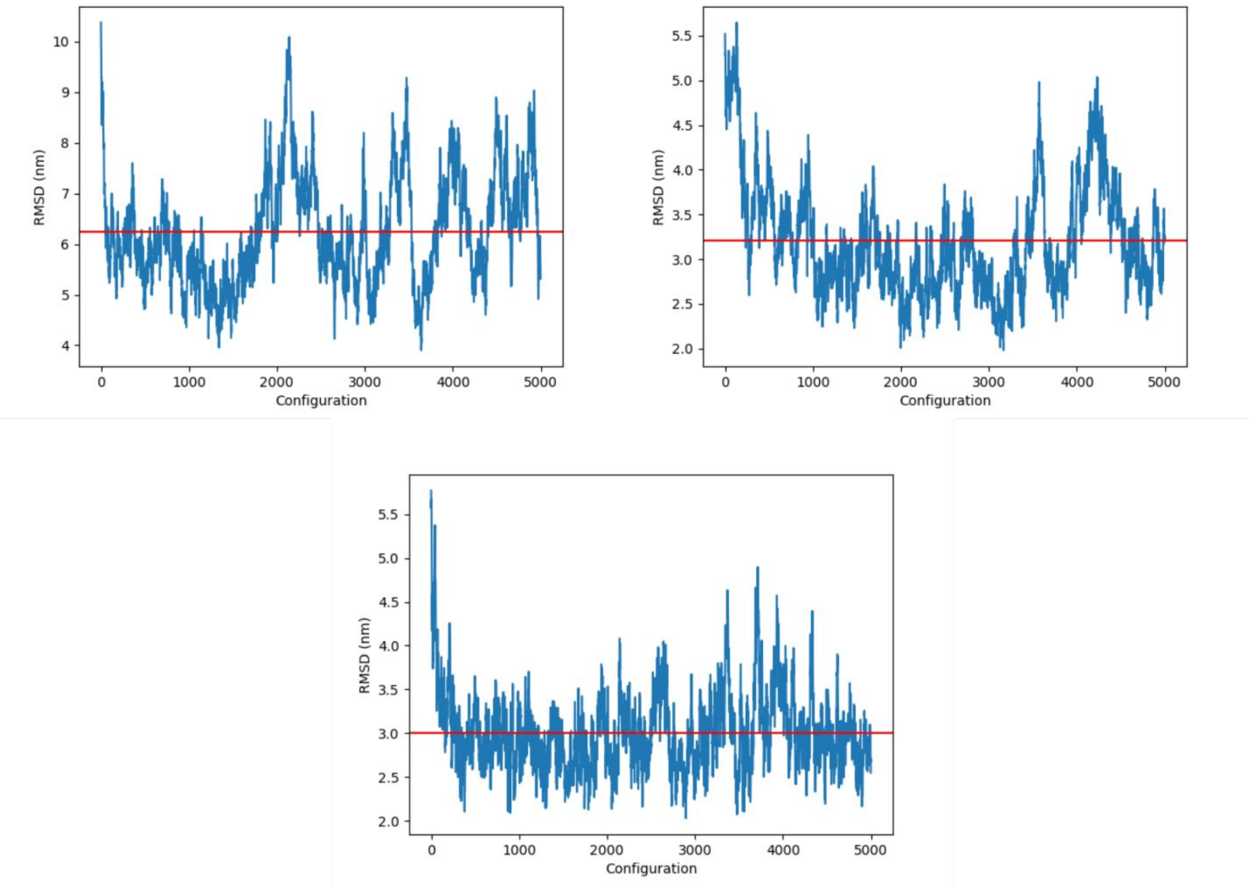

Supplementary Figure S24. Plot of the RMSD obtained from the oxDNA simulations for the non-reinforced rod, the reinforced rod and the vHelix hexagon rod.

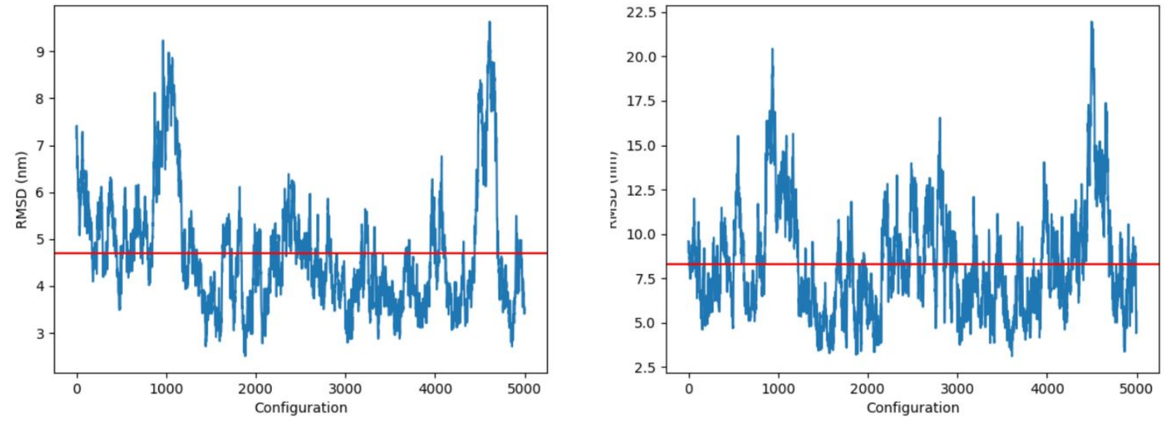

Supplementary Figure S25. Plot of the RMSD obtained from the oxDNA simulations for the reinforced part of the hybrid rod and for the non-reinforced part of the hybrid rod.

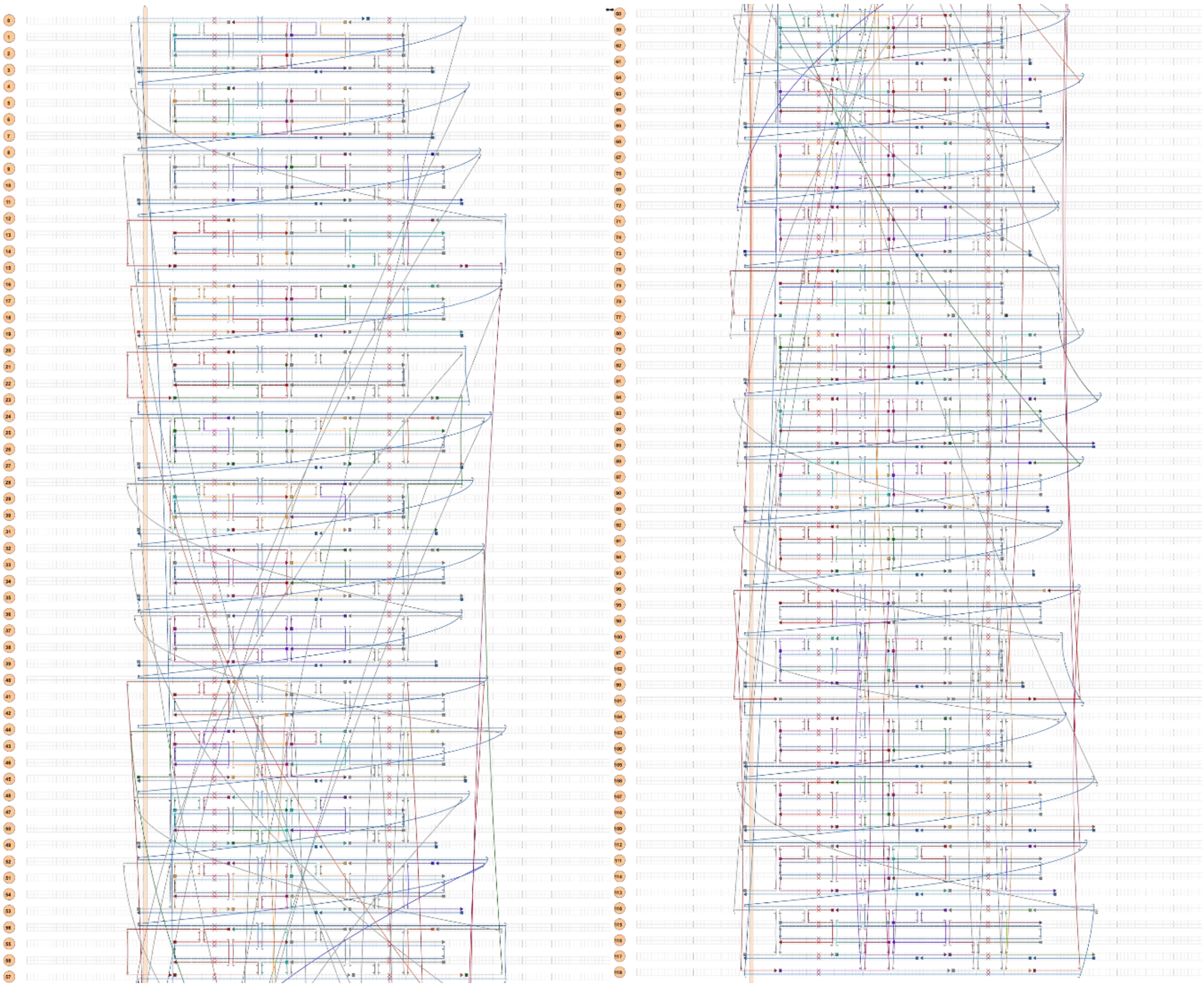

Supplementary Figure S26. caDNAno design of the reinforced icosahedron

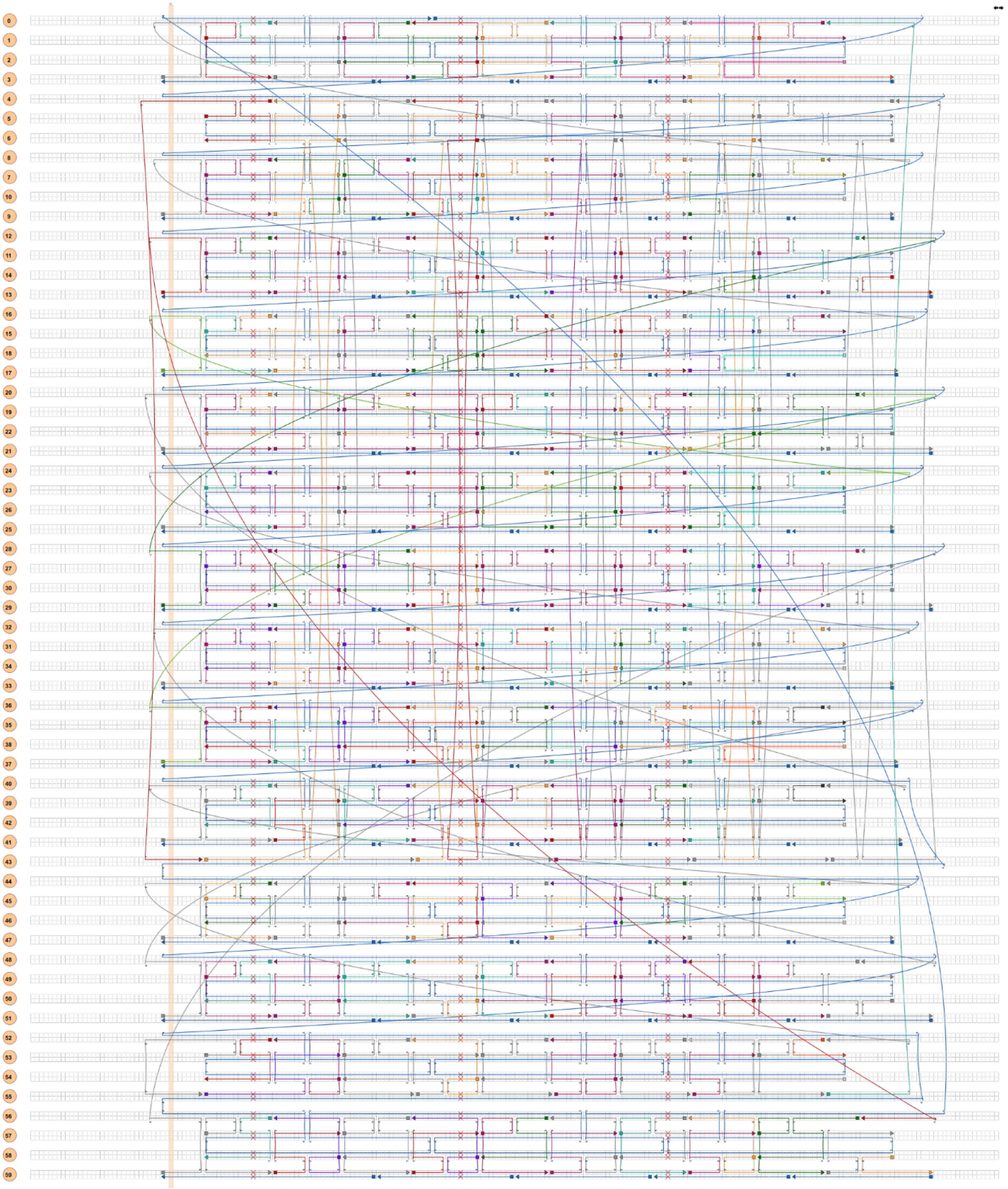

Supplementary Figure S27. caDNAno design of the reinforced pentagonal bipyramid

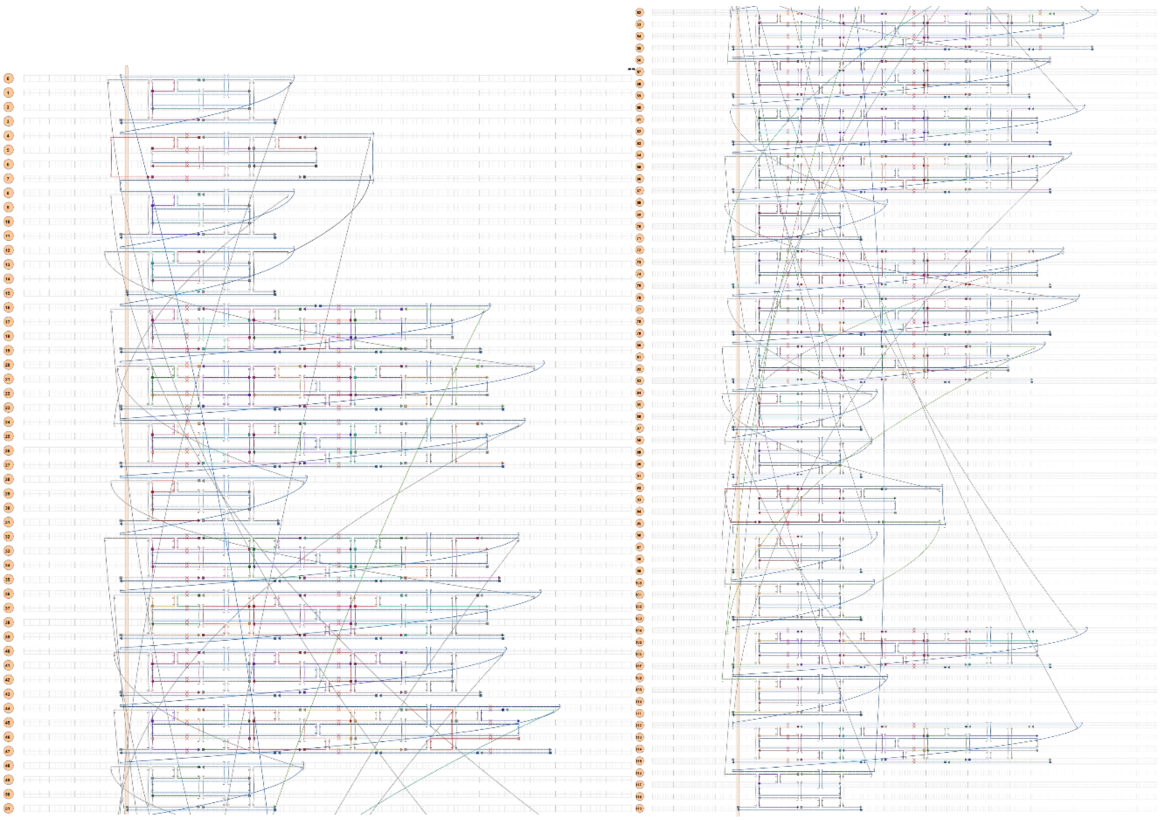

Supplementary Figure S28. caDNAno design of the reinforced rod

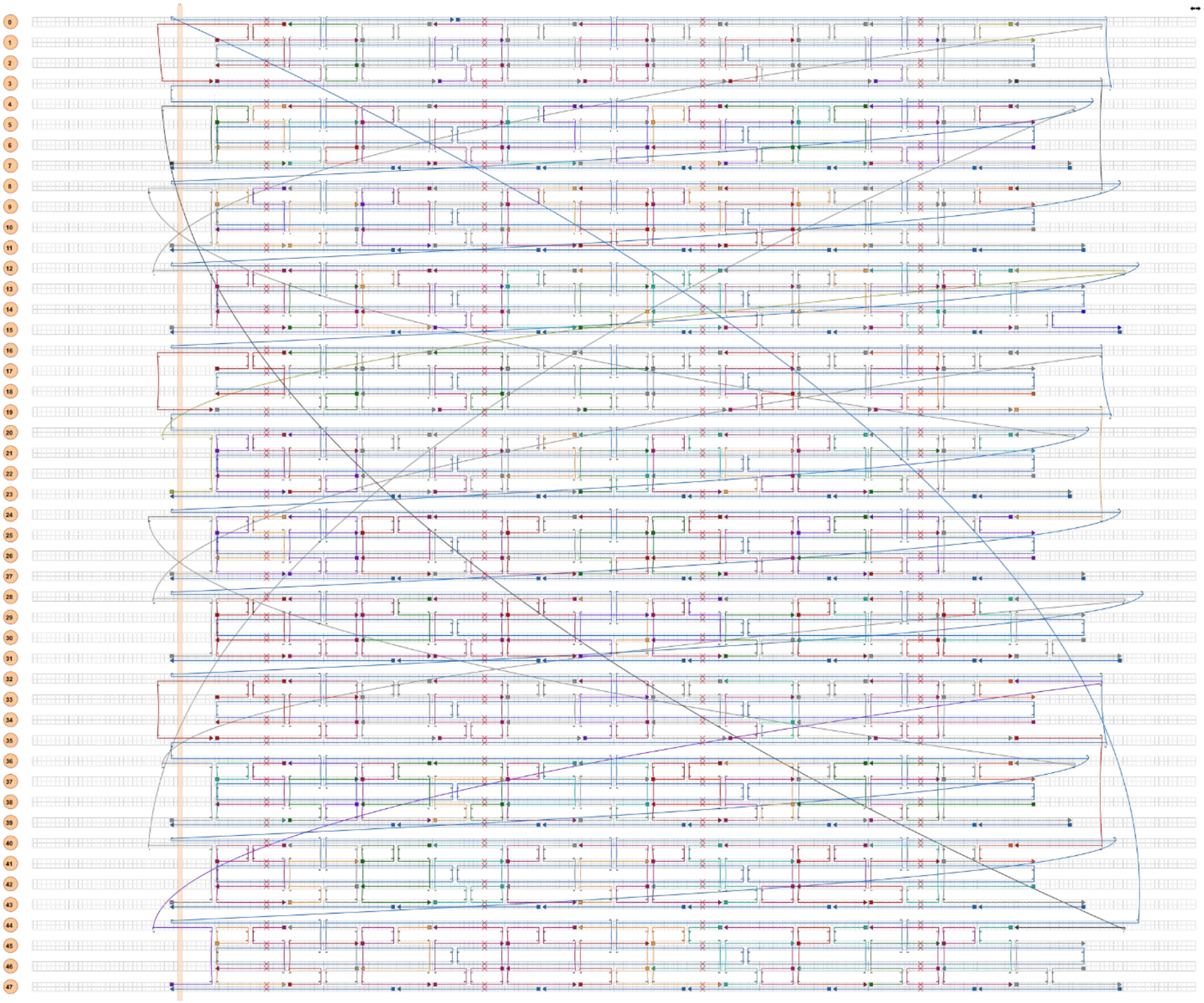

Supplementary Figure S29. caDNAno design of the reinforced hexagonal mesh

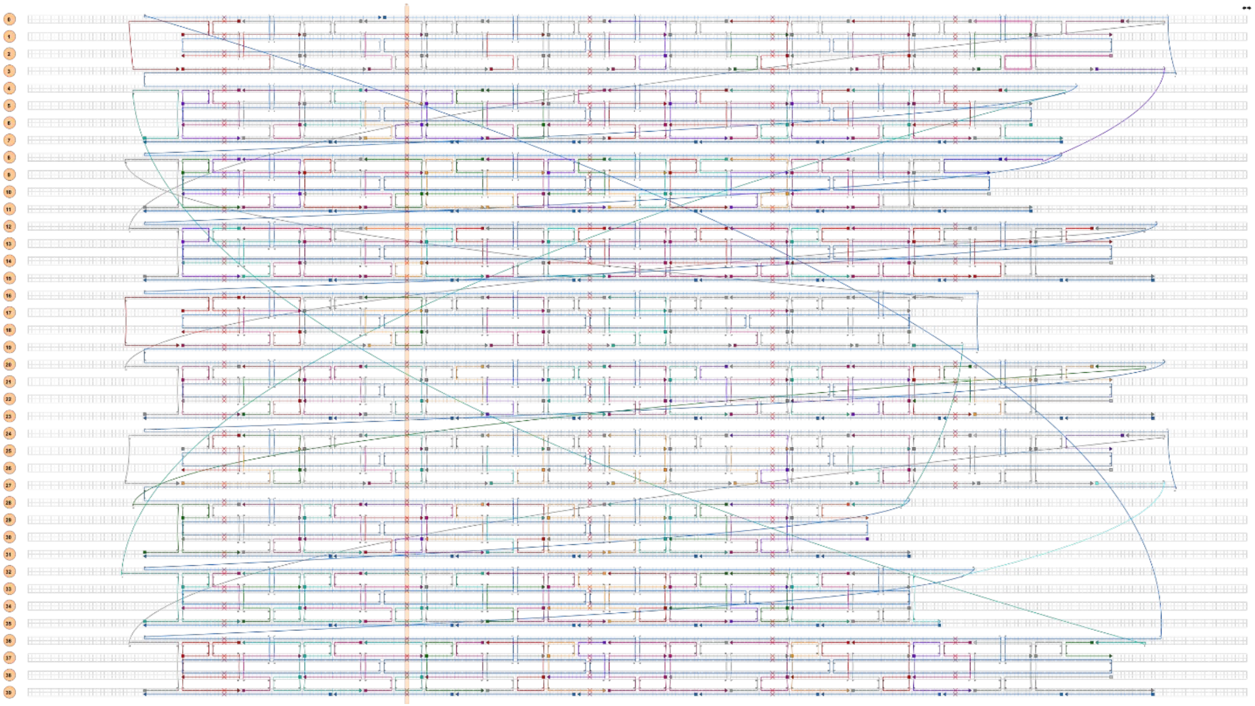

Supplementary Figure S30. caDNAno design of pentagonal mesh with edges of around 270 bp.

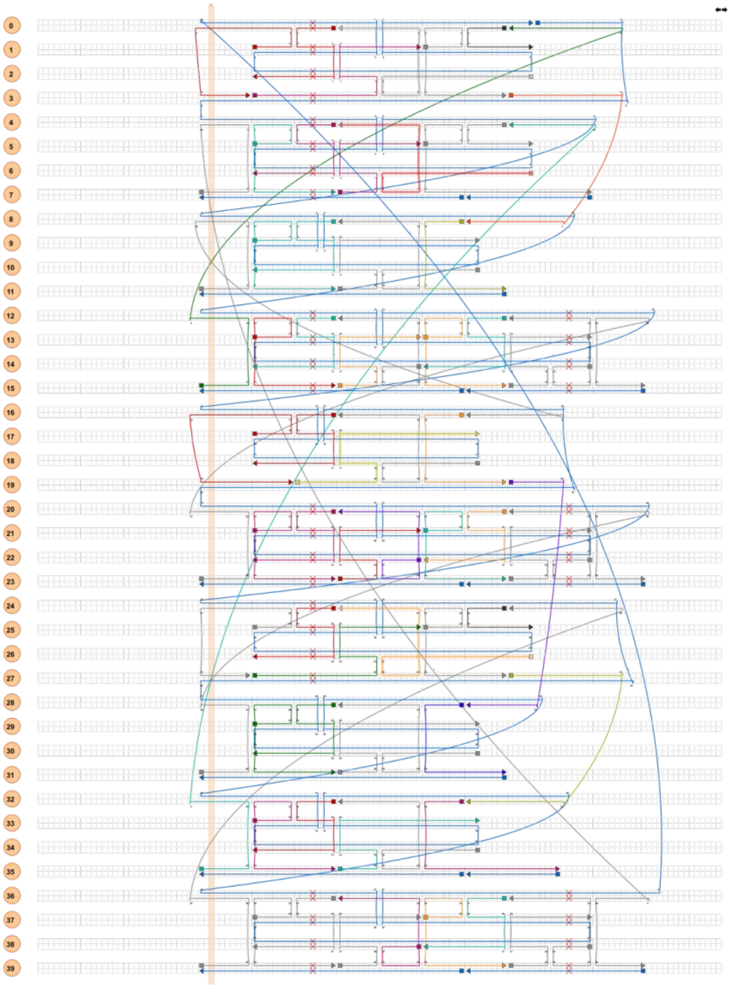

Supplementary Figure S31. caDNAno design of pentagonal mesh with edges of around 80 bp.

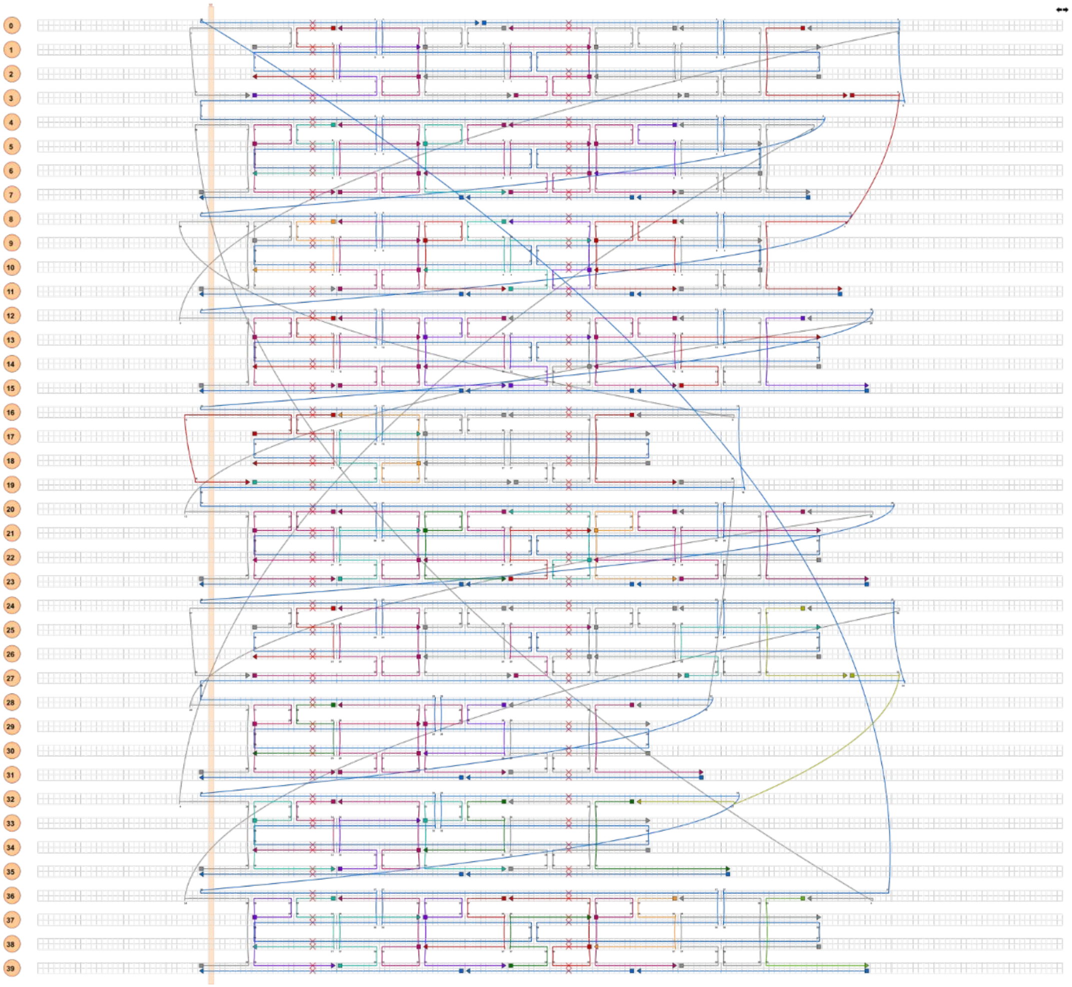

Supplementary Figure S32. caDNAno design of pentagonal mesh with edges of around 130 bp.

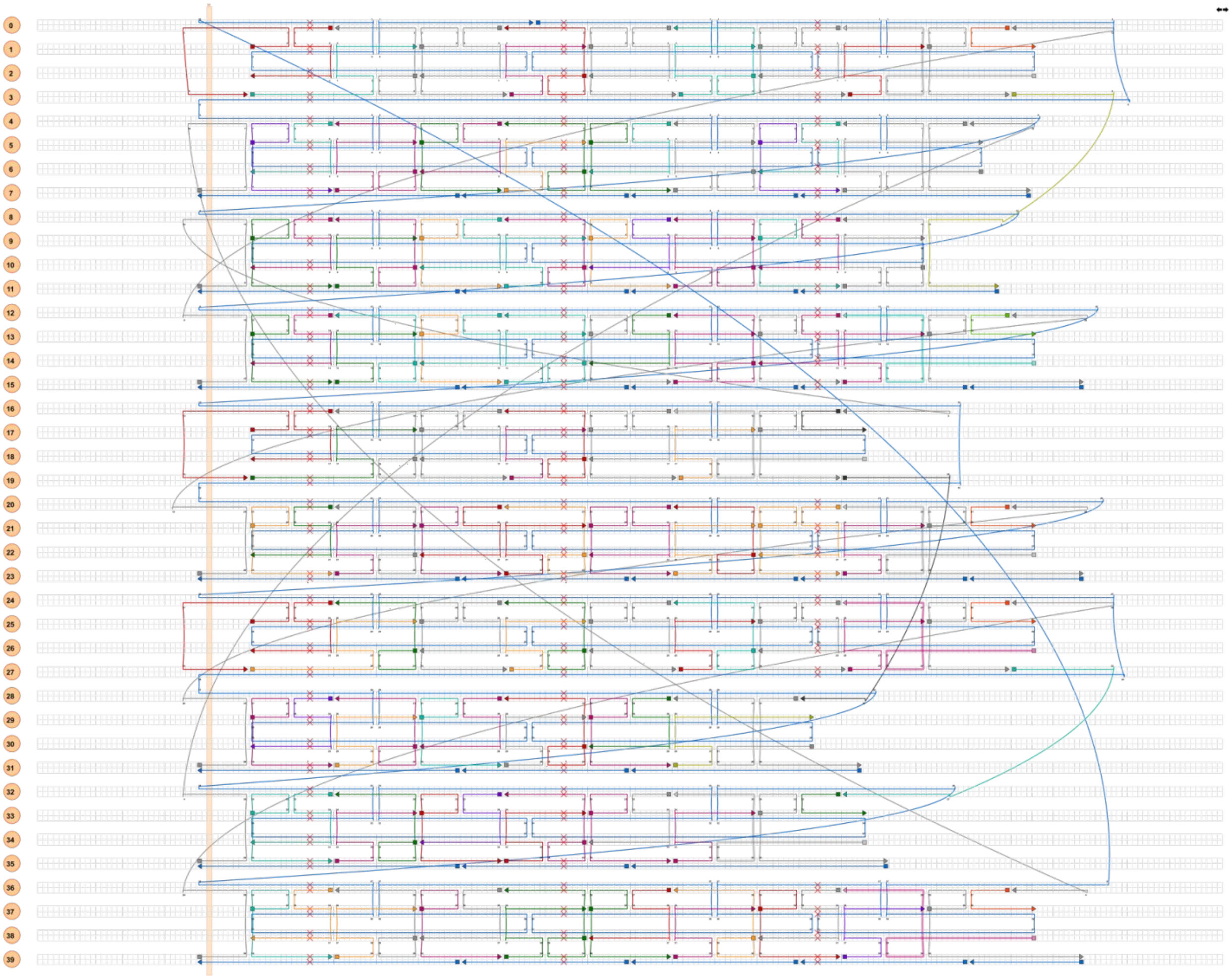

Supplementary Figure S33. caDNAno design of pentagonal mesh with edges of around 170 bp.

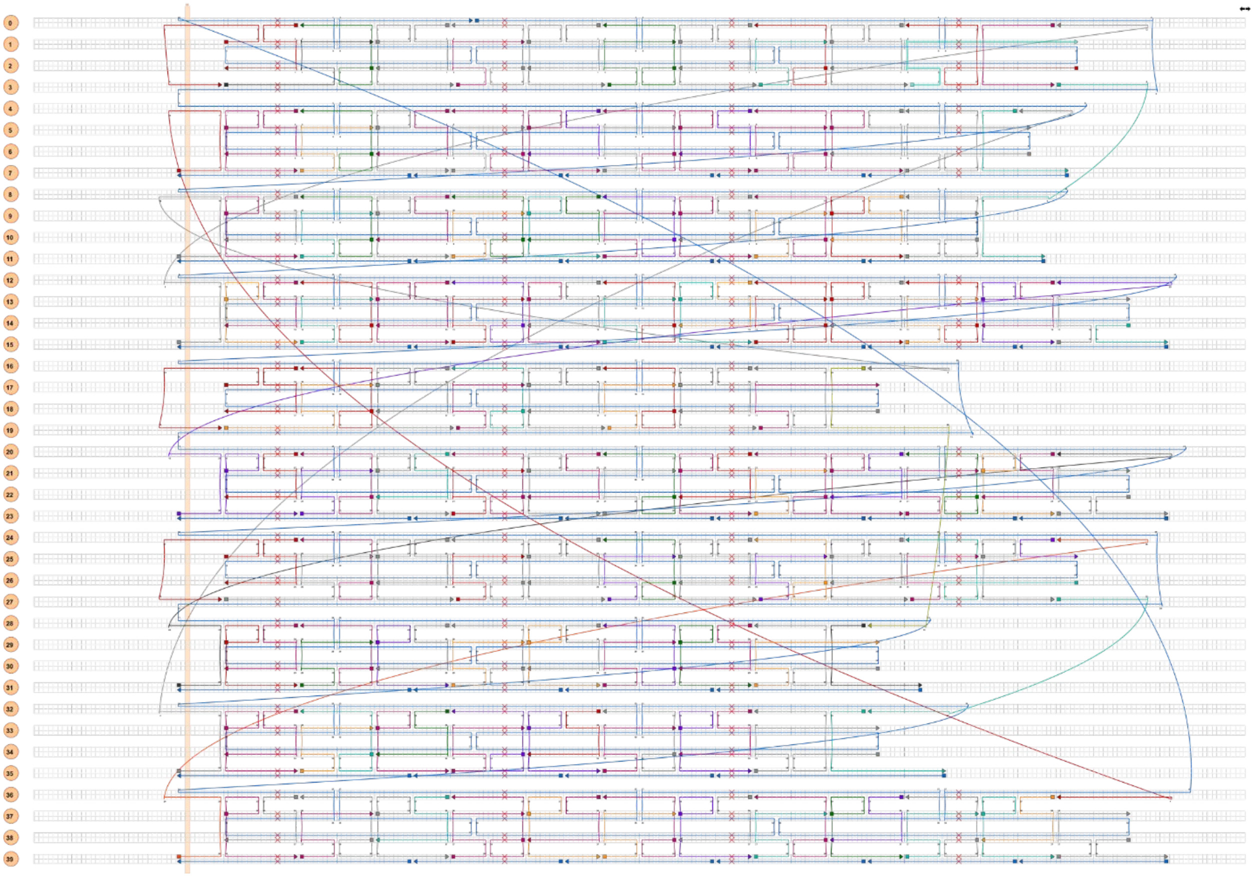

Supplementary Figure S34. caDNAno design of pentagonal mesh with edges of around 200 bp.

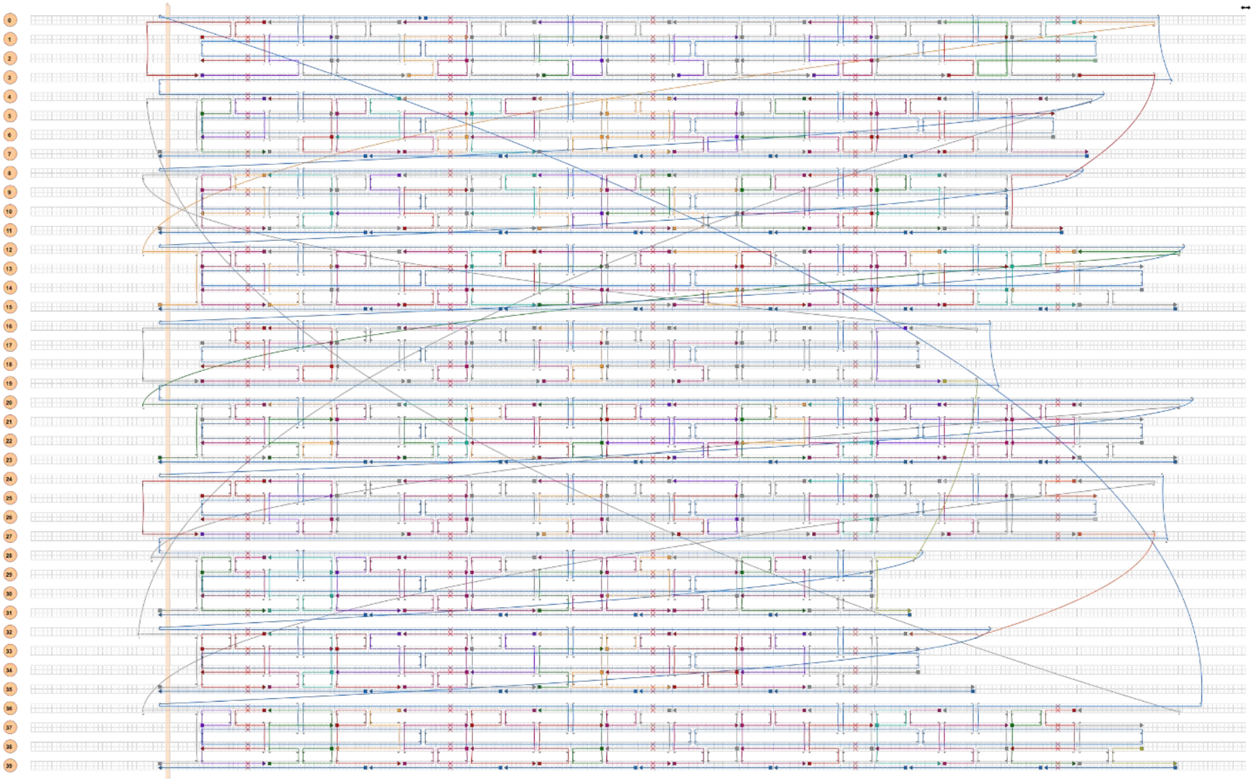

Supplementary Figure S35. caDNAno design of pentagonal mesh with edges of around 230 bp.

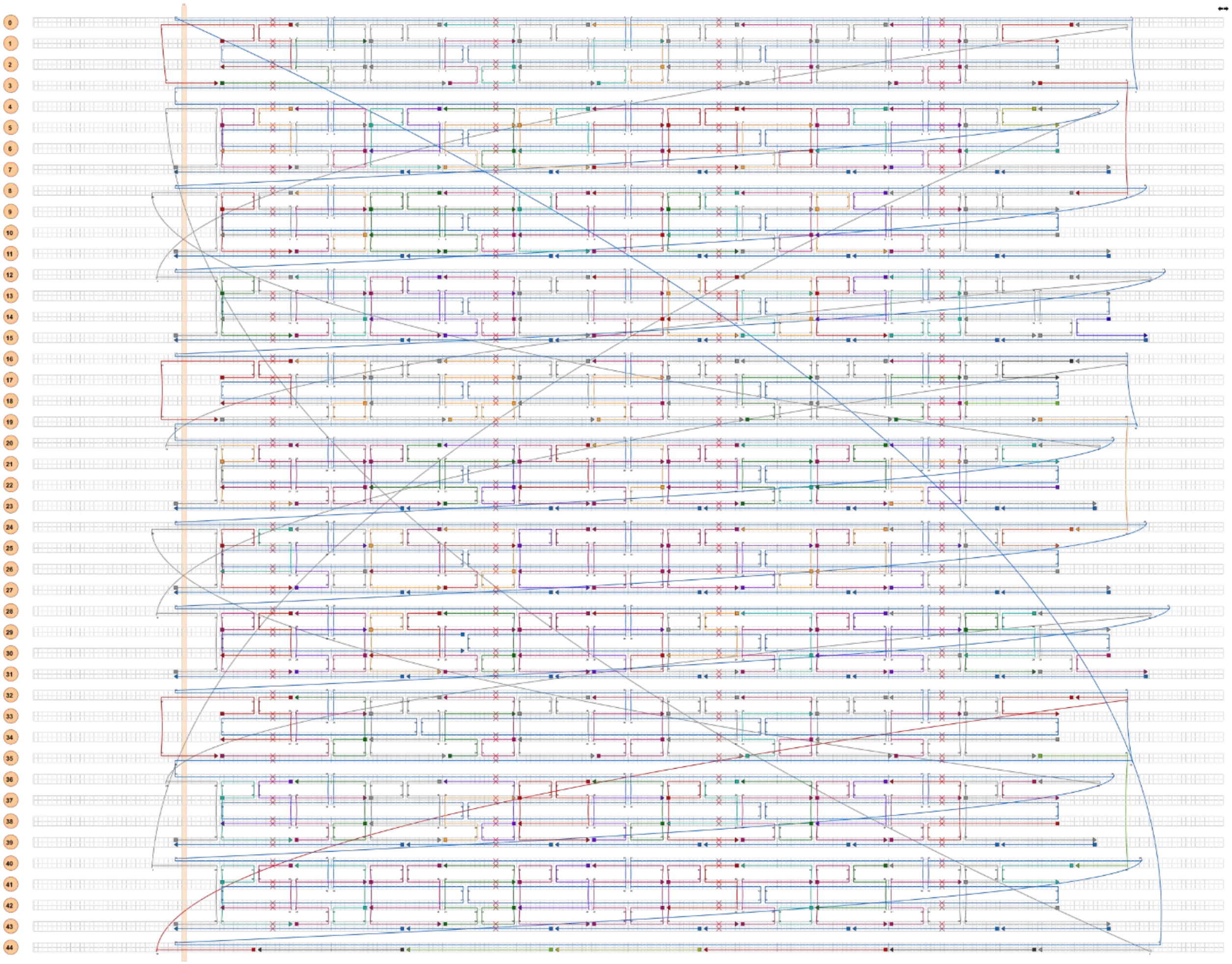

Supplementary Figure S36. caDNAno design of the hybrid hexagon.

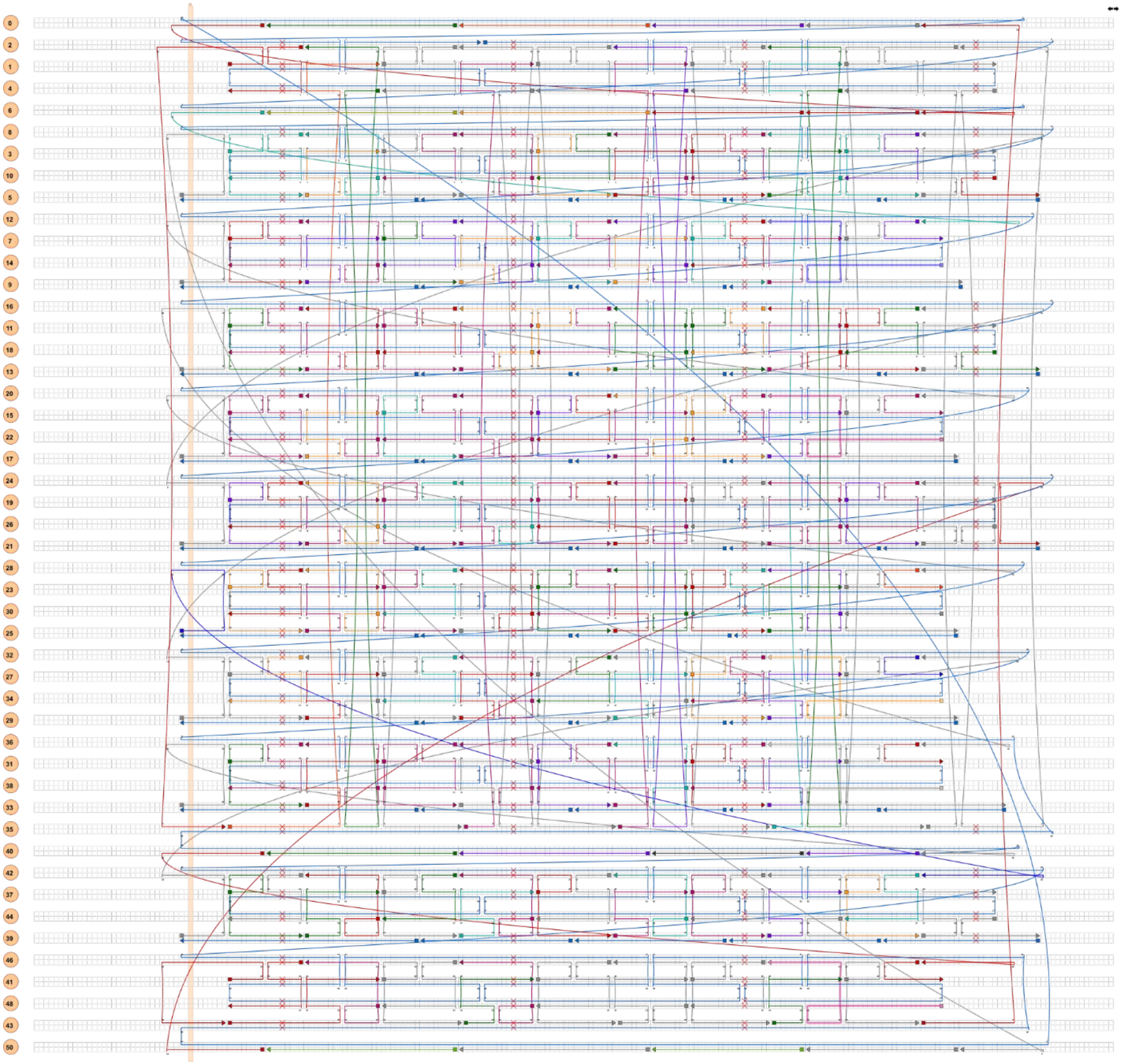

Supplementary Figure S37. caDNAno design of the hybrid pentagonal bipyramid.

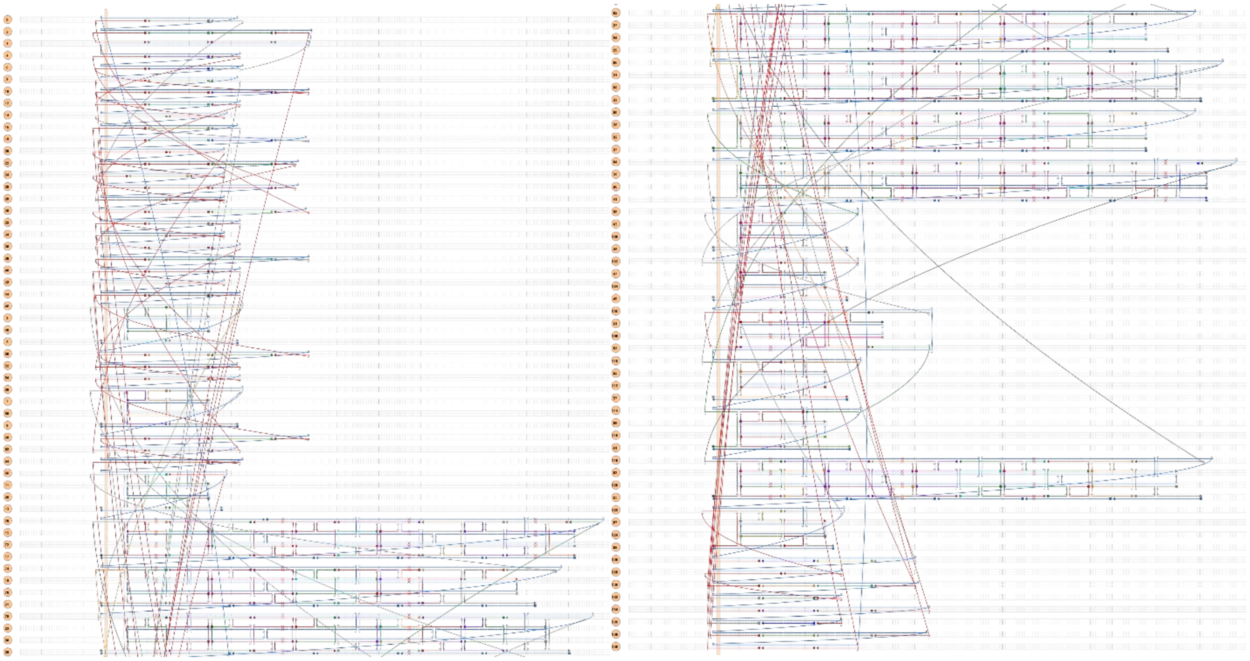

Supplementary Figure S38. caDNAno design of the hybrid rod.

Structure	Folding program	Salt concentration
Icosahedron	65°C for 15 min, from 53°C to 50°C at a rate of 60 min/°C, 4°C forever	22 mM $MgCl_2$
Pentagonal bipyramid	65°C for 15 min, from 53°C to 50°C at a rate of 60 min/°C, 4°C forever	18 mM $MgCl_2$
Reinforced rod	65°C for 15 min, from 53°C to 50°C at a rate of 60 min/°C, 4°C forever	22 mM $MgCl_2$
Flat hexagonal mesh	65°C for 15 min, 65°C to 60°C in 20 min, from 60°C to 40°C at a rate of 11 min/°C	22 mM $MgCl_2$
Flat pentagonal mesh Fig. 1	65°C for 15 min, 65°C to 60°C in 20 min, from 60°C to 40°C at a rate of 11 min/°C	22 mM $MgCl_2$
Flat pentagonal mesh 80 bp	65°C for 15 min, 65°C to 60°C in 20 min, from 60°C to 40°C at a rate of 11 min/°C	22 mM $MgCl_2$

Flat pentagonal 130 bp	65°C for 15 min, 65°C to 60°C in 20 min, from 60°C to 40°C at a rate of 11 min/°C or 16 hours program: 65 °C for 4 min, then 65 °C to 50 °C for 1 min per 0.7 °C, 50 °C to 35 °C for 1 h per 1 °C and 20 °C forever	22 mM $MgCl_2$
Flat pentagonal 170 bp	65°C for 15 min, 65°C to 60°C in 20 min, from 60°C to 40°C at a rate of 11 min/°C or 16 hours program: 65 °C for 4 min, then 65 °C to 50 °C for 1 min per 0.7 °C, 50 °C to 35 °C for 1 h per 1 °C and 20 °C forever	22 mM $MgCl_2$
Flat pentagonal 200 bp	65°C for 15 min, 65°C to 60°C in 20 min, from 60°C to 40°C at a rate of 11 min/°C or 16 hours program: 65 °C for 4 min, then 65 °C to 50 °C for 1 min per 0.7 °C, 50 °C to 35 °C for 1 h per 1 °C and 20 °C forever	22 mM $MgCl_2$
Flat pentagonal 230 bp	65°C for 15 min, 65°C to 60°C in 20 min, from 60°C to 40°C at a rate of 11 min/°C or 16 hours program: 65 °C for 4 min, then 65 °C to 50 °C for 1 min per 0.7 °C, 50 °C to 35 °C for 1 h per 1 °C and 20 °C forever	22 mM $MgCl_2$
Hexagon rod p7560	65°C for 15 min, 65°C to 60°C in 20 min, from 60°C to 40°C at a rate of 11 min/°C or 16 hours program: 65 °C for 4 min, then 65 °C to 50 °C for 1 min per 0.7 °C, 50 °C to 35 °C for 1 h per 1 °C and 20 °C forever	1x PBS

Hybrid Hexagon	65°C for 15 min, 65°C to 60°C in 20 min, from 60°C to 40°C at a rate of 11 min/°C	14 mM $MgCl_2$
Hybrid pentagonal bipyramid	65°C for 15 min, from 53°C to 50°C at a rate of 60 min/°C, 49°C to 40°C at a rate of 11 min/°C	20 mM $MgCl_2$
Hybrid rod	65°C for 15 min, from 51.4°C to 48.4°C at a rate of 60 min/°C, 4°C forever	18 mM $MgCl_2$

Supplementary Table 1. Folding conditions for the structures presented in this work.

Structure	Estimated yield from gel S12	Estimated yield from gel S13
Icosahedron	~30%	~80%
Pentagonal bipyramid	~45%	~85%
Reinforced rod	~30%	~60%
Flat hexagonal mesh	~30%	~70%
Flat pentagonal mesh Fig. 1	~5%	~20%

Supplementary Table 2. Estimated folding yield for the reinforced structures presented in Figure 1. The yield is estimated from the gels indicated in the table using Imagej.

Structure	Estimated yield from gel S14
Hybrid Hexagon	~30%
Hybrid pentagonal bipyramid	~25%
Hybrid rod	~40%

Supplementary Table 3. Estimated folding yield for the hybrid structures presented in Figure 4. The yield is estimated from the gel indicated in the table using Imagej.

(1) Benson, E.; Mohammed, A.; Gardell, J.; Masich, S.; Czeizler, E.; Orponen, P.; Högberg, B. DNA Rendering of Polyhedral Meshes at the Nanoscale. *Nature* **2015**, *523* (7561), 441–444. https://doi.org/10.1038/nature14586.

(2) Benson, E.; Mohammed, A.; Bosco, A.; Teixeira, A. I.; Orponen, P.; Högberg, B. Computer-Aided Production of Scaffolded DNA Nanostructures from Flat Sheet Meshes. *Angewandte Chemie* **2016**, *128* (31), 9015–9018. https://doi.org/10.1002/ange.201602446.

(3) Ke, Y.; Bellot, G.; Voigt, N. v.; Fradkov, E.; Shih, W. M. Two Design Strategies for Enhancement of Multilayer-DNA-Origami Folding: Underwinding for Specific Intercalator Rescue and Staple-Break Positioning. *Chem Sci* **2012**, *3* (8), 2587–2597. https://doi.org/10.1039/c2sc20446k.

(4) Ke, Y.; Douglas, S. M.; Liu, M.; Sharma, J.; Cheng, A.; Leung, A.; Liu, Y.; Shih, W. M.; Yan, H. Multilayer DNA Origami Packed on a Square Lattice. *J Am Chem Soc* **2009**, *131* (43), 15903–15908. https://doi.org/10.1021/ja906381y.

(5) Engelhardt, F. A. S.; Praetorius, F.; Wachauf, C. H.; Brüggenthies, G.; Kohler, F.; Kick, B.; Kadletz, K. L.; Pham, P. N.; Behler, K. L.; Gerling, T.; Dietz, H. Custom-Size, Functional, and Durable DNA Origami with Design-Specific Scaffolds. *ACS Nano* **2019**, *13* (5), 5015–5027. https://doi.org/10.1021/ACSNANO.9B01025/SUPPL_FILE/NN9B01025_SI_003.ZIP.
